# Curcumin Promotes A-beta Fibrillation and Reduces Neurotoxicity in Transgenic *Drosophila*

**DOI:** 10.1371/journal.pone.0031424

**Published:** 2012-02-13

**Authors:** Ina Caesar, Maria Jonson, K. Peter R. Nilsson, Stefan Thor, Per Hammarström

**Affiliations:** 1 IFM-Department of Chemistry, Linköping University, Linköping, Sweden; 2 IKE-Department of Clinical and Experimental Medicine, Linköping University, Linköping, Sweden; Consejo Superior de Investigaciones Cientificas, Spain

## Abstract

The pathology of Alzheimer's disease (AD) is characterized by the presence of extracellular deposits of misfolded and aggregated amyloid-β (Aβ) peptide and intraneuronal accumulation of tangles comprised of hyperphosphorylated Tau protein. For several years, the natural compound curcumin has been proposed to be a candidate for enhanced clearance of toxic Aβ amyloid. In this study we have studied the potency of feeding curcumin as a drug candidate to alleviate Aβ toxicity in transgenic *Drosophila*. The longevity as well as the locomotor activity of five different AD model genotypes, measured relative to a control line, showed up to 75% improved lifespan and activity for curcumin fed flies. In contrast to the majority of studies of curcumin effects on amyloid we did not observe any decrease in the amount of Aβ deposition following curcumin treatment. Conformation-dependent spectra from p-FTAA, a luminescent conjugated oligothiophene bound to Aβ deposits in different *Drosophila* genotypes over time, indicated accelerated pre-fibrillar to fibril conversion of Aβ_1–42_ in curcumin treated flies. This finding was supported by *in vitro* fibrillation assays of recombinant Aβ_1–42_. Our study shows that curcumin promotes amyloid fibril conversion by reducing the pre-fibrillar/oligomeric species of Aβ, resulting in a reduced neurotoxicity in *Drosophila*.

## Introduction

*Drosophila melanogaster* (*Drosophila*) has during the past decade emerged as a promising model system for Alzheimer's disease (AD) research [1], [2], [3]. Expression of various AD-related human proteins e.g., amyloid-β (Aβ) and Tau, in the *Drosophila* model system results in animals displaying many of the histological hallmarks of AD seen in humans [4], as well as a correlation of lifespan to aggregation propensity of the protein or peptide expressed [5] which surpasses that of corresponding rodent models. *Drosophila* Aβ models have also been used as platforms for pharmacological treatment assays by the putative aggregation inhibitor Congo red [3], designed native state stabilizers [6], and by genetic manipulations co-expressing molecular chaperones [7].

For several years, curcumin has been proposed to be a candidate drug for enhanced clearance of the toxic amyloids generated by the Aβ peptide. Curcumin has also been reported to have anti-inflammatory and anti-oxidative activity thus preventing tissue damage [8], [9], [10], [11], to reduce Aβ-induced toxicity [12], as well as to reduce microglia activation [13]. Many studies, *in vitro* and *in vivo*, have shown inhibition of Aβ oligomer [9] and fibril formation [9], [14], [15], [16] in the presence of curcumin, depending on the used assay. Curcumin appears to be a promiscuous AD-drug as it has also been shown to block Aβ toxicity *in vivo* through inhibition of Tau phosphorylation [11], [17], as well as regulating AβPP and BACE-1 transcription by interfering with copper ions [18] in cell cultures. Because of these promising results curcumin has been tested in humans as a drug candidate for AD [19]. The dominating view is that curcumin is an aggregation inhibitor, but recent studies of curcumin have shown that while it inhibits the oligomeric forms of Aβ, it accelerates the formation of Aβ fibrils [20]. Curcumin is a potent binder of amyloid deposits, making it a molecular candidate for histological staining in pathology [9], [21], [22], and it has been suggested as a useful derivate in combination with near-infrared imaging in living subjects [23]. In addition to these results, curcumin has also shown effects, mainly due to its anti-inflammatory properties, on other diseases such as rheumatoid arthritis, pancreatitis, cancer, osteoarthritis, and in some ocular as well as gastrointestinal conditions, such as ulcerative colitis [24]. Current drug development strategies include development of curcumin analogues with similar biological activity as curcumin, but with improved pharmacokinetic characteristics, including increased bioavailability and water solubility [25]. Amyloidogenic proteins are known to be able to assemble into multiple forms of amyloid-like structures *in vitro* in a process known as “amyloid fibril polymorphism” [26], [27] suggesting that several mechanisms may be involved in amyloid formation. Multiple fibrillation pathways will probably result in multiple forms of pre-fibrillary species, responsible for toxicity. The connection between these complex folding processes and curcumin is not clear.

Here, we have addressed the potency of curcumin in alleviating AD-like symptoms in transgenic *Drosophila* models of AD. To this end we used four different Aβ expressing *Drosophila* lines (*Aβ_1–40_* single transgene, *Aβ_1–42_* single transgene, *Aβ_1–42_* double transgene, and *Aβ_1–42 E22G_* single transgene) [3], and one human *Tau* expressing *Drosophila* line [1], all expressed in the *Drosophila* central nervous system (CNS) and eyes by the Gal4/UAS system [28]. The longevity and the locomotor activity of the different genotypes were measured relative to a control line. To detect amyloid formation *ex vivo*, we combined antibody staining with a luminescent conjugated oligothiophene, p-FTAA [29], as a marker for amyloid, and collected structural dependent spectra from the probe for different time points as the aggregation accelerates in the tissue. These results, combined with *in vitro* fibrillation of recombinant Aβ and quantification of the soluble and insoluble Aβ produced in the flies in absence or presence of curcumin, shows that curcumin does not inhibit amyloid formation. On the contrary, curcumin rather accelerates amyloid fibril conversion, and hence reduce the pre-fibrillary species of Aβ. This suggests that the reduction of pre-fibrillar species underlies the observed mitigated neurotoxicity in *Drosophila*.

## Results

### *Drosophila* longevity

**Figure 1:**
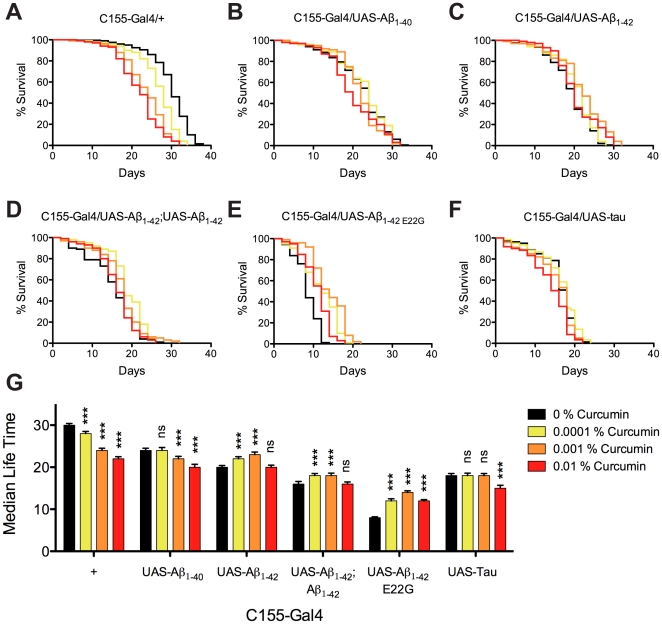
Curcumin affects Drosophila longevity as a function of genotype. Survival trajectories of transgenic *C155-Gal4 Drosophila* for different curcumin treatments indicated by: no curcumin added (black lines), 1, 10, and 100 µg curcumin per g yeast paste represented in yellow, orange, and red lines respectively. (A) Control flies showed a concentration dependent decrease in lifespan upon curcumin treatment. (B) Aβ_1–40_ expressing flies showed reduced survival times when fed intermediate and high curcumin concentrations. (C) Single insert Aβ_1–42_ expressing flies showed increased survival when treated with low and intermediate curcumin concentrations. (D) Double insert Aβ_1–42_ expressing flies showed an increased survival for low and intermediate curcumin concentrations. (E) Aβ_1–42 E22G_ expressing flies showed increased survival for all curcumin concentrations. (F) Survival of Tau expressing flies was unaffected at low and intermediate concentrations of curcumin, but revealed a shortened survival time at the high curcumin concentration. (G) Median survival time of all genotypes with no curcumin added represented in black bars, 1, 10, and 100 µg curcumin per g yeast paste represented in yellow, orange, and red bars respectively. Significance was compared using a paired student's t-test between untreated and treated with curcumin (ns: non-significant; *: P<0.05, **: P<0.01; ***: P<0.001).

To address the effects of curcumin upon AD-related symptoms in *Drosophila*, we initially fed various concentrations of curcumin (0–0.01% w/w in yeast paste) to the control flies. Rather unexpectedly, this however rendered a reduced lifespan. The reduced viability was curcumin concentration dependent, resulting in a decreased median survival time (T_1/2_) (Figure 1A). The Aβ_1–40_ expressing flies showed a decreased T_1/2_ compared to the control flies, and was only affected by the higher curcumin concentrations, also here resulting in a decreased life span. For the low concentration of curcumin, the lifespan was unaffected (Figure 1B). Curcumin feeding of the single inserted Aβ_1–42_ expressing flies showed a positive effect of the T_1/2_ at low and intermediate curcumin concentrations and no effect of the T_1/2_ on the highest curcumin concentration (Figure 1C). Curcumin feeding of the double inserted Aβ_1–42_ expressing flies showed a positive effect at low and intermediate concentrations of curcumin treatments. The lifespan for high curcumin concentration treated flies was not apparently different when compared to the untreated flies (Figure 1D). All curcumin treatments of the Aβ_1–42 E22G_ expressing flies showed a substantial positive effect upon curcumin treatment. The greatest observed effect was found on 0.001% curcumin treatment of the Aβ_1–42 E22G_ expressing flies, which increased the T_1/2_ by 75% compared to untreated flies (Figure 1E). Curcumin feeding of the Tau expressing flies rendered no effect on survival at low concentrations, but a toxic effect on high concentrations of curcumin (Figure 1F). The median survival time (Figure 1G) of all transgenes and curcumin concentrations displayed a clear effect upon curcumin treatment for genotypes having the strongest phenotype. The toxic effect on curcumin treatment was balanced with the rescuing effect of genotypes having a mild phenotype. The T_1/2_ for all genotypes and curcumin concentrations are summarized in Table S1. The results from the longevity assay were essentially mirrored in a conventional climbing assay with a reduced climbing shifted of 1–2 days prior to death (Figure S1) as reported in previous studies [30].

### *Drosophila* locomotor activity

**Figure 2:**
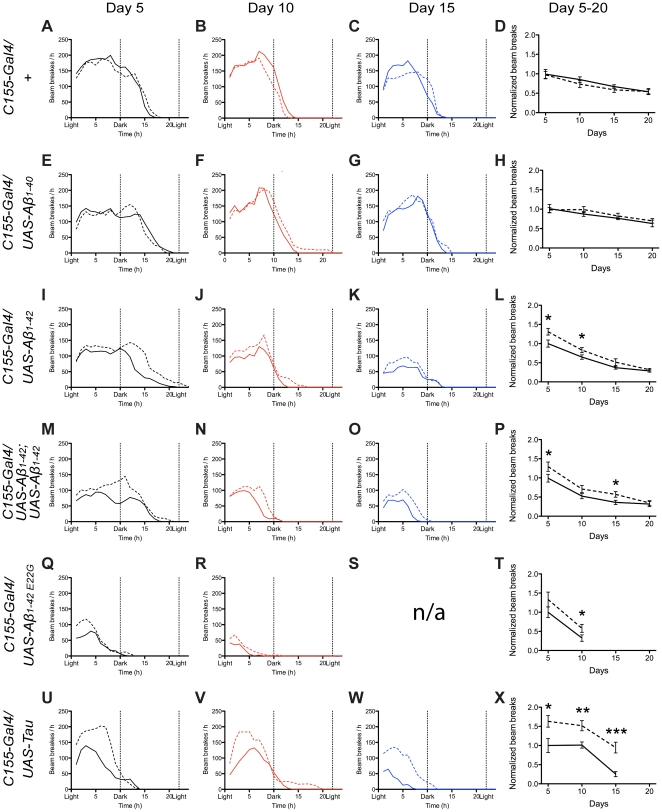
Curcumin affects *Drosophila* locomotor activity as a function of genotype. Activity traces (DAM2 system) shown for flies without (solid line) and with (dotted line) curcumin treatment (10 µg per g of yeast paste), performed 5, 10, 15, and 20 days after eclosion. (A–D) Control flies showed an activity decrease with increased age. The decreased number of total beam brakes upon increased age was caused due to the shortened number of active hours. (E–H) Aβ_1–40_ expressing flies showed no improvement in activity upon curcumin treatment. (I–L) In young flies, single insert Aβ_1–42_ expressing flies showed a higher number of beam breaks and a longer continuation of activity upon curcumin treatment. The effect of curcumin decreased with increasing age. (M–P) Double insert Aβ_1–42_ expressing flies showed an activity enhancement upon curcumin treatment, but the effect of curcumin decreased with increasing age. (Q–T) Aβ_1–42 E22G_ expressing flies showed an increased number of beam breaks during the initial hours of monitoring during curcumin treatment, but the number of active hours was not prolonged for 5 days old flies. A small curcumin induced increase in activity was observed for 10 day old flies. (U–X) Tau expressing flies, at all ages, showed a great enhancement of locomotor activity during the first hours of experiments upon curcumin treatment. Significance was compared using a paired student's t-test between untreated and treated with curcumin (ns: non-significant; *: P<0.05, **: P<0.01; ***: P<0.001).

The DAM2 system automatically counts the number of beam breaks for flies walking in a horizontal tube over a period of 24 hours [31]. This setup allowed for characterization of the locomotor and behavior rhythms of *Drosophila*. Comparing the locomotor activity of the flies without curcumin treatment at day 5 (c.f. Figure 2A, E, I, M, Q, U), there were obvious differences in the activity and circadian rhythm between the different genotypes. The DAM2 system hence appeared more sensitive for assaying early signs of neurological impairment compared to the conventional climbing assay (*cf.* Figure 2 with Figure S1). Studies of continuous curcumin treatment of flies at the intermediate concentration (0.001%) was performed at different days of aging, with 5 days increments. Control flies showed slight activity deterioration upon curcumin treatment. Overall, the locomotor activity of the flies decreased with increasing age, but the number of beam breaks per hour was almost consistent during the first hours of the assay. The decreased total number of beam brakes upon increased age was caused by the shortened number of active hours (Figure 2A–D). Aβ_1–40_ expressing flies showed no activity improvement upon curcumin treatment (Figure 2E–H). Single insert Aβ_1–42_ expressing flies showed a higher number of beam breaks and a continuation of activity during a larger number of hours upon curcumin treatment for flies at 5 and 10 days (Figure 2I–L). The effect of curcumin showed a tendency of decreasing with age (Figure 2L). Double insert Aβ_1–42_ expressing flies showed an activity enhancement upon curcumin treatment for flies of all ages (Figure 2M–P). Also here the effect of curcumin showed a tendency of declining with age (Figure 2P). The Aβ_1–42 E22G_ expressing flies showed a severely decreased locomotor activity already at day 5 compared to control flies (c.f. Figure 2A and 2Q). Curcumin treated Aβ_1–42 E22G_ expressing flies at day 5 showed an increased number of beam breaks per hour during the first hours, but the number of active hours was not significantly enhanced (Figure 2Q). A small but significant increase in activity was observed for 10 days old Aβ_1–42 E22G_ expressing flies treated with curcumin (Figure 2R). The activity assay was performed with the unusual population of the Aβ_1–42 E22G_ expressing flies surviving two days longer than T_1/2_ of the untreated Aβ_1–42 E22G_ expressing flies. No activity assay was performed for Aβ_1–42 E22G_ expressing flies at ages beyond 10 days, due to their short lifespan. Tau expressing flies appear severely affected in their locomotor activity, and interestingly showed a large enhanced activity during the first hours upon curcumin treatment (Figure 2U–X). The activity enhancement was sustained with increasing age, which was different from the Aβ transgenes (Figure 2X). Notably, the Tau expressing flies showed the greatest increase in locomotor activity of all genotypes upon curcumin treatment, but as described above, these flies did not show any significant effect in the lifespan or in climbing behavior upon curcumin treatment (Figure 1 and. Figure S1).

### Histological analysis of amyloid deposits in the *Drosophila* brain over time

**Figure 3:**
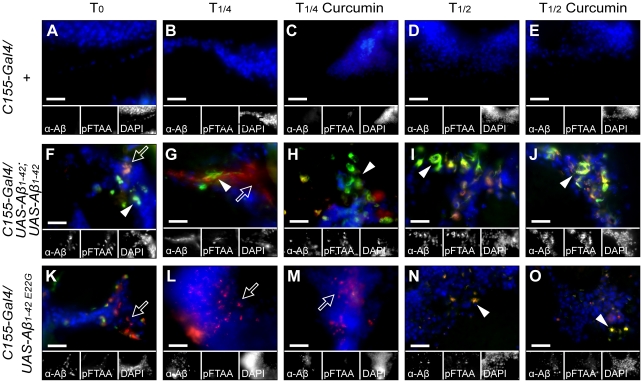
Curcumin affects the brain amyloid deposition histology patterns as a function of *Drosophila* genotype. Micrographs of *Drosophila* brains taken with 100× objective showing fluorescence from cell nuclei by DAPI (blue), amyloid aggregates by p-FTAA (green) and Aβ by αAβ-antibody (red). (A–E) Control flies with and without treatment with curcumin at day 0, day 10, and day 20, showed no antibody or p-FTAA binding species in the brain tissue. (F–J) Double insert Aβ_1–42_ expressing flies showed extensive amyloid staining by p-FTAA, with several long extended fibrillar aggregates at all time points. Curcumin treatement accelerated p-FTAA positive (amyloid aggregate) conversion at young age (c.f. Figure 3G and H). The amount of detectable aggregates increased with age. DAPI staining from regions with widespread p-FTAA-positive aggregates were decreased. (K–O) Aβ_1–42 E22G_ expressing flies at day 0, day 5, and day 10, showed a spot-like staining from both p-FTAA and the Aβ specific antibody, but a weaker p-FTAA staining was apparent (c.f. Fig. 3 I and N). Some irregular nuclei were visible from the DAPI staining. Day 0 and day 5 flies showed protein aggregates detectable with the antibody and to some extent by p-FTAA. The same staining pattern was reveled irrespective of curcumin treatment. Filled arrowheads show p-FTAA positive amyloid aggregates and open arrows indicate αAβ-antibody positive aggregates (diffuse Aβ accumulation). Scale bars in insets represent 20 µm.

*Drosophila* brains were investigated by fluorescence microscopy using combined antibody and amyloid specific luminescent conjugated oligothiophene (LCO), p-FTAA, for the presence of amyloid deposition [29]. Images shown in Figure 3 and Figure S2 represent parts of brains where protein deposition was obvious. The histological analysis described below was based on visual inspection of 20 flies of each genotype and is summarized in Table 1 before the median life span (T_1/2_) for each genotype. Control flies with and without curcumin, displayed no antibody or LCO binding in the brain tissue (Figure 3A–E), but unspecific staining was seen in the retina and exoskeletal head capsule, as described before [32]. However, the double inserted Aβ_1–42_ expressing flies showed extensive amyloid staining, with several long extended fibrillar aggregates found already in newly eclosed flies (Figure 3F). The amounts of aggregates visible by LCO staining increased with increasing age (Figure 3F, G, I). Some staining differences was observed in ten day old flies, where the untreated flies displayed stronger antibody staining and decreased LCO staining compared to treated flies (Figure 3G, H, Table 1). The staining pattern from ten-day-old curcumin treated flies did not differ from twenty-day-old flies, independent of treatment (Figure 3G–J). DAPI staining from regions with a high load of LCO-positive aggregates was decreased, suggesting neuronal loss. The Aβ_1–42 E22G_ expressing flies displayed a spot-like staining from both LCO and Aβ antibody independent of age and treatment, but a weaker LCO staining than that displayed for wild type single and double inserted Aβ_1–42_ expressing flies (Figure 3K–O). The number of small aggregates detectable with the LCO increased only slightly with increasing age. The antibody staining decreased with increasing age. Aβ_1–40_ expressing flies (Figure S2A–E) displayed weak and restricted LCO and antibody amyloid staining surrounding the nuclei in twenty-day-old flies. Amyloid staining in the Aβ_1–40_ expressing flies has never earlier been reported. At earlier time points, LCO amyloid staining was only shown to a minute extent, but antibody staining was detected in the areas with high amounts of cell nuclei (Table 1). There was no difference between the untreated and curcumin treated flies. Single insert Aβ_1–42_ expressing flies (Figure S2F) showed antibody staining, as well as some LCO staining, in both newly eclosed flies and in untreated ten day old flies. Interestingly, ten-day-old flies treated with curcumin displayed a different staining pattern than the untreated flies, where LCO positive aggregates were found in most regions of the brain that contained cell bodies. The intensity of the LCO staining appeared enhanced in the curcumin treated flies (Figure S2G, H). In twenty-day-old flies the difference appeared to be retained between untreated and curcumin treated flies. Both untreated and curcumin treated flies had a strong amyloid staining, predominantly surrounding the nuclei, but regions of large aggregates were more commonly observed in curcumin treated flies than in untreated flies (Figure S2I, J, Table 1). The Tau expressing flies showed few LCO and antibody-binding aggregates in young flies (Figure S2K–M). At the age of 15 days, LCO binding aggregates were detected in approximately one out of five flies. There was no difference in staining upon curcumin treatment (Figure S2N, O, Table 1). The antibody staining increased between day 0 and day 10, but decreased in day 15 brains. The aggregates were mostly found in regions were no or few cell nuclei were visible. The visual inspection of brain histology rendering assessment of LCO and antibody reactivity respectively is summarized in Table 1, before the T_1/2_ of lifespan for each genotype.

**Table 1:** Phenotypes of *C155-Gal4/UAS-Aβ* and *C155-Gal4/UAS-tau* Alzheimer Disease Models of *Drosophila* treated and untreated with 0.001% (w/w) of curcumin.

GenotypeCurcumin +/−	control		Aβ_1–40_		Aβ_1–42_		Aβ_1–42_; Aβ_1–42_		Aβ_1–42 E22G_		tau	
	−	+	−	+	−	+	−	+	−	+	−	+
**Survival**	30±0.4	24±0.5	24±0.5	22±0.6	20±0.4	23±0.6	16±0.6	18±0.6	8±0.2	14±0.4	18±0.2	18±0.5
**Climbing**	28±0.7	24±0.5	20±06	22±0.6	18±0.5	23±0.6	16±0.6	16±0.5	8±0.2	8±0.2	8±0.2	8±0.35
**Activity day 5** a	1.000	0.974	0.860	0.831	0.661	0.914	0.495	0.719	0.187	0.269	0.409	0.689
**Activity day 10** a	0.823	0.705	0.749	0.851	0.431	0.564	0.260	0.367	0.057	0.104	0.418	0.659
**Activity day 15** a	0.671	0.592	0.655	0.719	0.242	0.327	0.160	0.272	n.a.	n.a	0.089	0.378
**Activity day 20** a	0534	0.538	0.534	0.591	0.177	0.213	0.144	0.176	n.a.	n.a	n.a.	n.a.
**α- ** ***Aβ or Tau*** ** reactivity** b	−	−	+^d^	+^d^	++	++	+++	++	++	++	++^d^	++^d^
**p-FTAA reactivity** c	−	−	+^d^	+^d^	+	++	++	+++	+	+	+^d^	+^d^

a Defined as total number of beam brakes for 16 flies in the DAM2 system over 24 h. Flies were reared at 29°C and assayed at the same temperature.

b Protein deposition load determined by visual inspection with a fluorescence microscope of immunofluorescence of antibody detection, of histological sections of *Drosophila* heads at day 10 for *C155-Gal4/UAS-Aβ_1–40_*, *C155-Gal4/UAS-Aβ_1–42_*, *C155-Gal4/UAS-Aβ_1–42_;UAS-Aβ_1–42_*, *C155-Gal4/UAS-tau*; and day 5 for *C155-Gal4/UAS-Aβ_1–42 E22G_*. At least 20 fly heads were assayed for each genotype. +++, extensive; ++, intermediate; + detectable; −, not detected; n.a., not assayed.

c Aggregate deposition load determined by visual inspection with a fluorescence microscope of p-FTAA and immunofluorescence of histological sections of *Drosophila* heads at day 10 for *C155-Gal4/UAS-Aβ_1–40_*, *C155-Gal4/UAS-Aβ_1–42_*, *C155-Gal4/UAS-Aβ_1–42_;UAS-Aβ_1–42_*, *C155-Gal4/UAS-tau*; and day 5 for *C155-Gal4/UAS-Aβ_1–42 E22G_*. At least 20 fly heads were assayed for each genotype. +++, extensive; ++, intermediate; + detectable; −, not detected; n.a., not assayed.

d Not all flies showed protein aggregates, given is the load when protein aggregates were detected.

### Spectral analysis of amyloid formation in *Drosophila* over time

**Figure 4:**
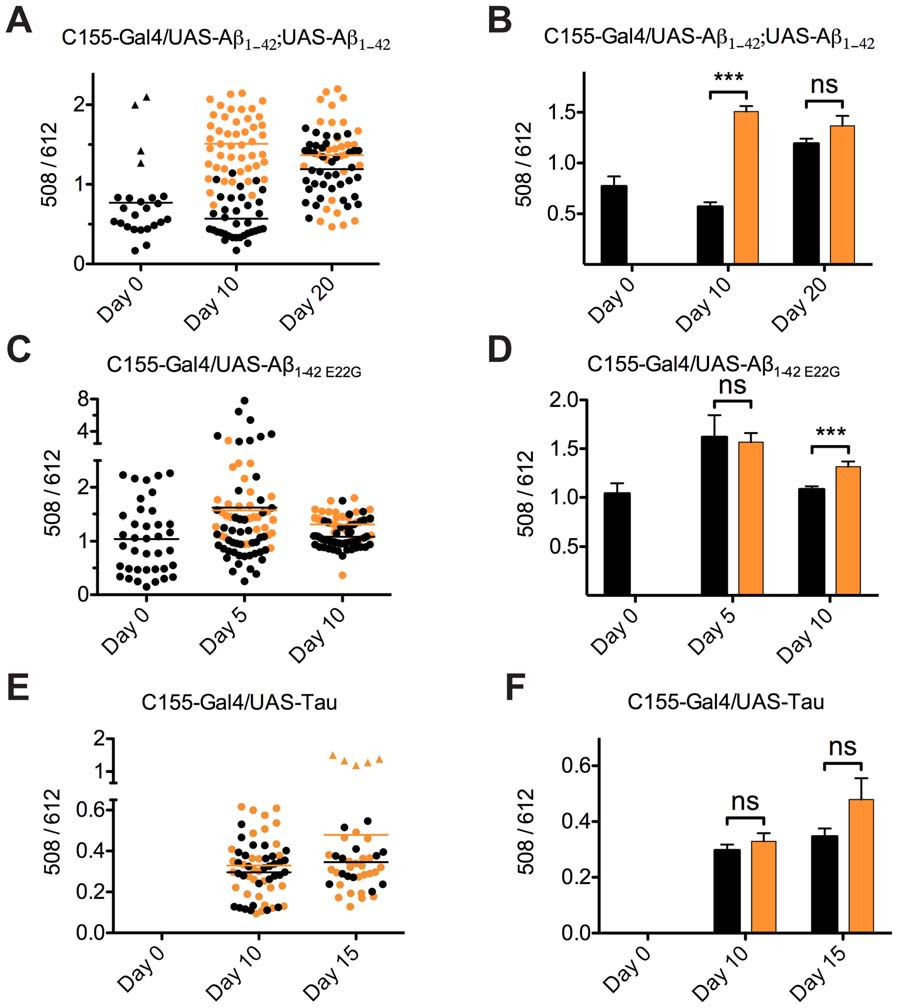
Curcumin accelerates aggregate to fibril conversion in *Drosophila* brains. Amyloid fibrillation index as individual spectral shift (A, C, and E) and mean spectral shift with standard deviation (B, D, and F) over time, in presence (orange) or absence (black) of curcumin treatment for Aβ and Tau expressing flies. (A and B) Newly eclosed double insert Aβ_1–42_ expressing flies had variable emission spectra, resulting in a wide variation in the 508/612 ratios. Some aggregates with the distinct amyloid feature were also detected (marked with triangles in the graph) in young flies. The double insert Aβ_1–42_ expressing flies displays a higher amyloid fibrillation index after ten days of curcumin treatment, indicating a more organized fibril formation due to curcumin ingestion. Untreated flies did not display an increased Aβ-fibrillation index at day 10. At day 20, the overall amyloid fibrillation index had increased, but there were no differences in the amyloid-fibrillation index between untreated and treated flies. (C and D) The Aβ_1–42 E22G_ expressing flies showed a rather high amyloid fibrillation index already in newly eclosed flies. Both treated and untreated flies have higher Aβ-fibrillation index at day 5, but at day 10 the Aβ-fibrillation index was lower, especially for untreated flies. (E and F) The Tau expressing flies showed more red shifted emission spectra than the Aβ genotypes, with a higher contribution from the 612 nm emission (560 nm excitation) (Figure S3), resulting in a lower amyloid fibrillation index. Note the different scale of the y-axis compared to A–D. A few aggregates with elevated amyloid fibrillation index were detected as marked by triangles in the graph. Overall the trend was the same as for Aβ expressing flies but the statistical analysis revealed that no significant differences were observed for the Tau expressing flies upon curcumin treatment. No spectra could be analyzed in Tau expressing flies at day 0, due to low detection rate and minute aggregates, resulting in low spectral intensities. (ns: non-significant; *: P<0.05, **: P<0.01; ***: P<0.001).

Sections stained with the LCO, p-FTAA [29], were analyzed by hyperspectral imaging. As expected from previous imaging experiments in *Drosophila* models, the p-FTAA emission spectrum of compact plaques differed depending on genotype [32]. We herein assessed if curcumin treatment rendered a conformational difference of the amyloid deposits within the *Drosophila* transgenics. To this end we employed spectral mixing and quantification of the fluorescence intensities at 508 nm (excitation at 405 nm) and 612 nm (excitation at 560 nm) (Figure S3) as described previously in detail for transgenic APP mice [33]. We used this parameter as an amyloid fibrillation index. A high ratio of 508/612 nm was interpreted as a well structured amyloid fibril whereas a low ratio was interpreted as alternative conformational states of the aggregates. A spectral shift over time, in presence or absence of curcumin treatment (0.001%) was clearly observable for the double inserted Aβ_1–42_ expressing flies (Figure 4A and 4B). Newly eclosed flies had few aggregates detectable with p-FTAA. The aggregates had variable emission spectra, resulting in a wide variation in the amyloid fibrillation index (508/612 ratio values). Some aggregates with the distinct amyloid feature were also detected already at day 0 (Figure 4A, triangles). The Aβ aggregates within the double insert Aβ_1–42_ expressing flies displayed a higher amyloid fibrillation index, after ten days of curcumin treatment, indicating that curcumin promoted a more rapid conversion into more well organized fibrils. Untreated flies did not display the increased amyloid fibrillation index at day 10. At day 20, on the other hand, there were no differences in the amyloid fibrillation index between untreated and treated flies. Both groups of flies displayed a high amyloid fibrillation index indicating well organized Aβ-fibrils. An extended amyloid fibrillation index in two dimensions (employing also the 543 nm peak) was also employed for the double inserted Aβ_1–42_ expressing flies. This analysis further supported the notion that curcumin treatment promoted amyloid fibrillation into well organized structures (Figure S4). Control experiments, performing spectral imaging of curcumin treated flies without p-FTAA staining, did not result in any spectral images that were above noise level (Figure S5). Control experiments, where curcumin was used as a histological labeling agent, did not result in any significant fluorescence in the spectral imaging (Figure S5), ruling out that the spectral imaging results were influenced by curcumin fluorescence.

Protein aggregates within aged Aβ_1–42 E22G_ expressing flies was previously shown to display a slightly more red shifted emission spectrum compared to wild type double insert Aβ_1–42_ expressing flies [32], as well as in aged mice [33]. This was evident also using the double excitation hyper spectral imaging method employed above (c.f. 10 day Aβ_1–42 E22G_ expressing flies and 20 day wild type flies, Fig. 4A and 4C). The emission spectra of the Aβ_1–42 E22G_ expressing flies differed from the double insert Aβ_1–42_ expressing flies. The wild type double insert Aβ_1–42_ expressing flies showed a clear double peak of 508 nm and 543 nm, whereas the Aβ_1–42 E22G_ expressing flies only had a shoulder peak at 508 nm for late stage aggregates [32]. Because the peak at 508 nm increased for double insert Aβ_1–42_ expressing flies with increased age and increased fibril formation, it indicates that the Aβ_1–42 E22G_ expressing flies do not produce as well ordered aggregates as the wild type double insert Aβ_1–42_ expressing flies. Usually the spectra of the Aβ_1–42 E22G_ expressing flies was red shifted compared to the double insert Aβ_1–42_ expressing flies, and displayed the shoulder peak at 510 nm and the distinct second peak at 545 nm. Interestingly, the Aβ_1–42 E22G_ expressing flies showed an elevated amyloid fibrillation index in 5 day old flies (Figure 4C and D) compared to the amyloid fibrillation index at either 0 or 10 day old flies. Even so, no extended fibrous deposits were visible in the histological staining of Aβ_1–42 E22G_ expressing flies (Figure 3K–O). Both curcumin treated and untreated flies displayed indifferently the elevated Aβ-fibrillation index at day 5. At day 10 the amyloid fibrillation index was lower for untreated flies as in the case for wild type expressing flies at day 10 (Fig. 4C, D), albeit with smaller absolute differences than for the double inserted Aβ_1–42_ expressed flies at this age.

The Tau expressing flies showed significantly more red shifted emission spectra than either Aβ genotypes as reported before [32] (Figure S6). The red shifted spectra resulted in consistently lower amyloid fibrillation index (508/612 nm ratios) at both analyzed time points (Figure 4E and F). Although the same trend was found as for the single inserted Aβ_1–42_ expressing flies no significant spectral difference was observed for the Tau expressing flies upon curcumin treatment. Due to low detection rate and small aggregates, resulting in low spectral intensities, no spectra could be analyzed in Tau expressing flies at day 0.

### Quantification of Aβ-peptide in aged and curcumin treated *Drosophila*

The concentration of Aβ is the primary factor determining aggregation rates and likely also influences Aβ conformation within the formed aggregates. The effect of treating flies with curcumin on the levels of Aβ accumulation was assessed by the Meso Scale Discovery (MSD) immunoassay, measuring soluble (in Hepes buffer, pH 7.3) and insoluble Aβ (homogenate dissolved in Hepes buffer, pH 7.3 containing 5 M GuHCl) concentrations in the brains of flies. Two different lines of flies – double inserted Aβ_1–42_ expressing flies and Aβ_1–42 E22G_ expressing flies – were examined at three different time points (day 0, 10 and 20 after eclosion, for double insert Aβ_1–42_ expressing flies, and day 0, 5, and 10 after eclosion, for Aβ_1–42 E22G_ expressing flies).

**Figure 5:**
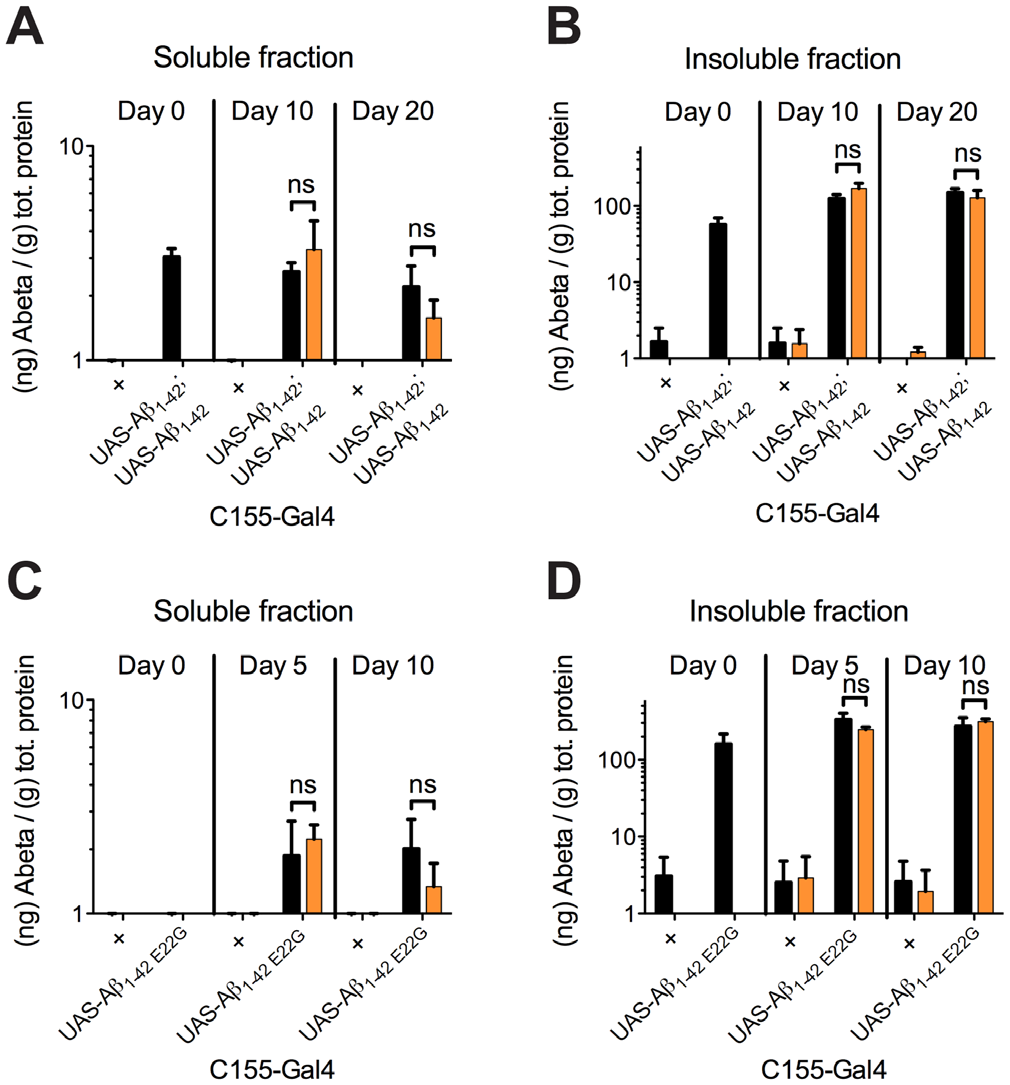
The amount of Aβ-peptide accumulation in *Drosophila* brain is not affected by curcumin. Quantification of Aβ-peptide in aged flies was performed using the Meso Scale Discovery (MSD) immunoassay. Soluble and insoluble Aβ concentrations for untreated flies (black bars) and curcumin treated flies (orange bars). (A) The double insert Aβ_1–42_ expressing flies showed an increased signal of Aβ compared to controls at all time points in the soluble fraction. There was no significant difference in the curcumin treated or untreated flies. (B) The double insert Aβ_1–42_ expressing flies showed an increased signal, at all time points, of Aβ compared to controls. The insoluble fraction accounted for the majority of Aβ, but there was no significant difference in Aβ concentration between untreated flies and curcumin treated flies. (C) Aβ_1–42 E22G_ expressing flies showed an increased signal of Aβ compared to controls at day 5 and day 10 in the soluble fraction. There was no significant difference in soluble Aβ of curcumin treated flies, when compared to untreated flies. (D) The Aβ_1–42 E22G_ expressing flies showed an increased signal of Aβ when compared to controls at all time points in the insoluble fraction, but there was no significant difference in Aβ concentration between untreated flies and curcumin treated flies. Error bars denote the mean standard deviation as deduced from triplicate samples in three independent experiments. (ns: non-significant; *: P<0.05, **: P<0.01; ***: P<0.001).

As anticipated, control flies displayed little signal of Aβ regardless of treatment with curcumin or not (Figure 5A–D, Table S3, Table S4). For the Aβ expressing flies the amount of total Aβ showed no significant difference between the untreated flies and the curcumin treated flies at either time point. Moreover, the fraction of soluble Aβ was not altered by curcumin treatment. Notably, the fraction of soluble Aβ was below 5% of the total Aβ for all genotypes at all time points. We hence concluded that treatment with curcumin does not shift the total amount of Aβ or the distribution of soluble versus insoluble Aβ. This finding supports the observation that curcumin does not decrease Aβ deposition. It is important to note that any increase in insoluble Aβ due to treatment would be difficult to detect with this assay, because >95% of Aβ was insoluble in untreated flies.

### Direct evidence for Aβ_1–42_ binding to curcumin *in vitro*

**Figure 6:**
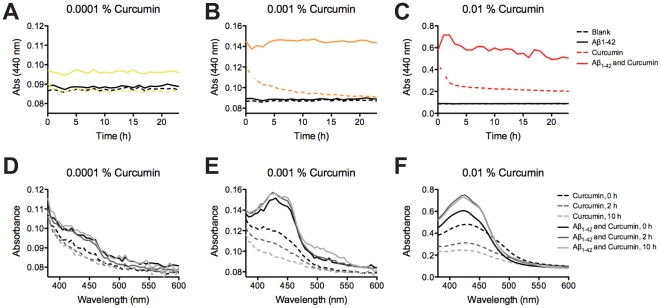
Direct evidence for Aβ_1–42_ binding *in vitro*. (A–C) Curcumin degradation at neutral pH (PBS) solution over time assessed by absorbance at 440 nm for solutions containing 0.0001% (yellow), 0.001% (orange), and 0.01% (red) (w/v) curcumin. Solid lines and dotted lines represented in presence and in absence of 10 µM Aβ_1–42_ peptide respectively. Black lines represents background absorbance of buffer (dotted line) and 10 µM Aβ_1–42_ peptide (solid line) in the absence of curcumin. (D–F) Curcumin absorbance spectra for the corresponding curcumin containing samples as those described in A–C at 0 h, 2 hours and 10 hours of incubation colored in gray scale.

It is well established that curcumin in solution at neutral pH is rapidly degraded [34], and this can also be observed by ocular inspection of an aqueous curcumin solution (not shown). In our formulation of curcumin in yeast paste for the fly food we did not note (by eye) a decrease in yellow coloration over time. We also noted that in the presence of Aβ_1–42_, curcumin solutions remained yellow over time. This is in contrast to observations of curcumin dissolved alone in PBS [34]. An absorbance assay was performed at 29°C to quantify this effect. In the absence of Aβ_1–42_ we observed a rapid decline in absorbance at 440 nm over time, with a half time of 1.5–3 h, depending upon curcumin concentration (Figure 6A–C). In the presence of Aβ_1–42_ this decline in absorbance was abolished at curcumin concentrations below that of Aβ_1–42_. In addition, we observed a noticeable red shifted absorbance spectrum of curcumin in the presence of excess Aβ_1–42_, supporting the notion of sequestered curcumin molecules in a hydrophobic environment (Figure 6D, E). Importantly, these results substantiate two important findings: i) Aβ_1–42_ directly binds curcumin (evident by the spectrochromic absorbance shift) and ii) Aβ_1–42_ sequesters curcumin from degradation (evident by the preserved curcumin absorbance over time).

### *In vitro* aggregation of Aβ_1–42_ in the presence of curcumin

**Figure 7:**
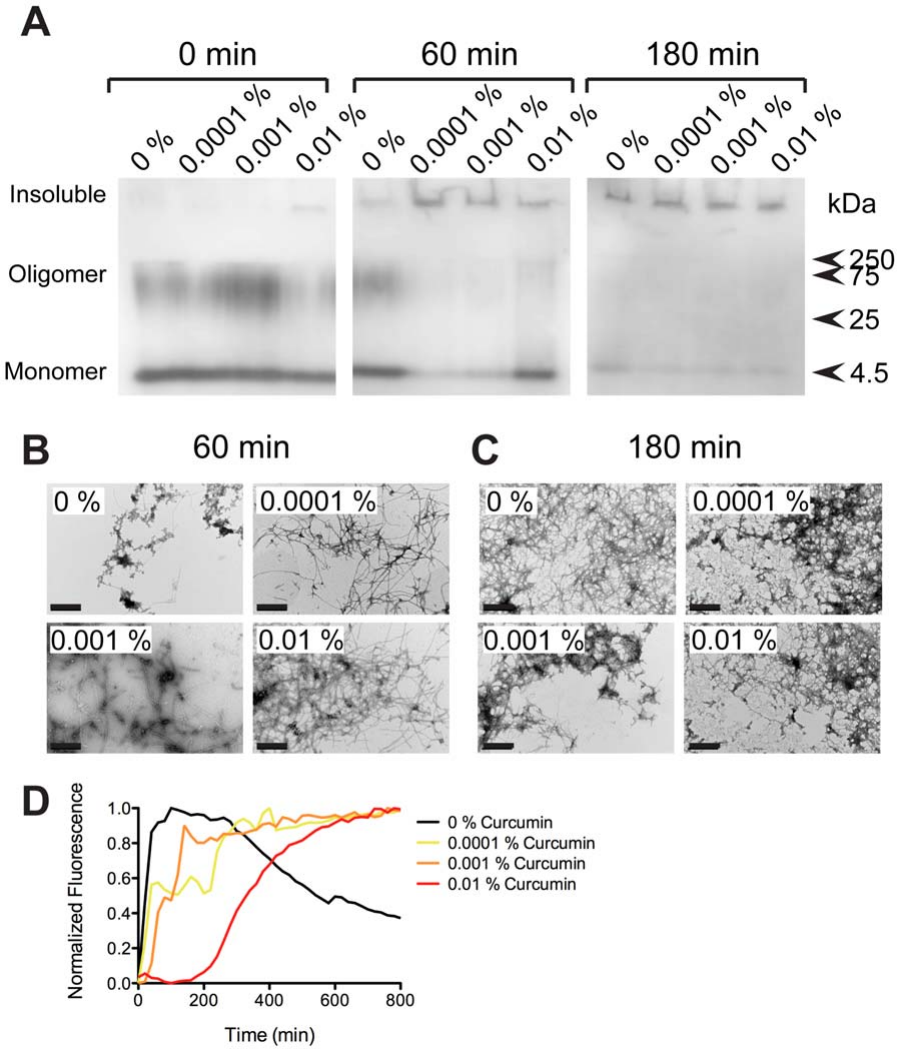
*In vitro* fibril conversion of Aβ_1–42_ is accelerated by curcumin. Aliquots of fibrillation reactions (10 µM Aβ_1–42_ in 10 mM phosphate pH 7.5, 37°C, agitation) were assayed at 0, 60 and 180 min. (A) Native-PAGE separation was interpreted as differential separation of monomeric, oligomeric and insoluble Aβ_1–42_. Lower populations of oligomeric species and elevated amounts of insoluble Aβ_1–42_ was observed in curcumin containing samples. (B) Transmission electron micrographs obtained at 60 minutes in the absence of curcumin revealed the presence of morphologically disordered aggregates with associated amyloid fibrils, whereas all concentrations of curcumin revealed the presence of amyloid fibrils. (C) At 180 minutes all samples contained extensively fibrillated Aβ_1–42_. Scale bars represent 100 nm. (D) p-FTAA fluorescence kinetics assaying formation of prefibrillar Aβ_1–42_ aggregates in the presence of vehicle (2% EtOH) and/or 0.0001, 0.001, and 0.01% (w/v) curcumin (0.27–27 µM curcumin). The concentration dependent suppression of the rapid prefibrillar phase, suggests accelerated conversion of Aβ_1–42_ to amyloid fibrils in the presence of curcumin. Absence of curcumin is represented with black line, 0.0001, 0.001, and 0.01% (w/v) curcumin is represented in yellow, orange, and red lines respectively.

Next, to compare our data with previous reports (e.g. [9]), we performed *in* vitro studies to address the effect of curcumin on the molecular aggregation behavior of Aβ_1–42_. Samples were assayed using native PAGE Western blotting, transmission electron microscopy (TEM), and p-FTAA fluorescence. Native PAGE separation of freshly dissolved Aβ_1–42_ showed the presence of monomeric Aβ_1–42_ and a diffuse band representing soluble oligomeric Aβ_1–42_ for all samples (Figure 7A). Native PAGE separation performed of the samples from the *in vitro* fibrillation at 60 minutes displayed an enhancement of insoluble Aβ_1–42_ in presence of curcumin, when compared to the control incubated with vehicle (1% ethanol). The enhanced band of insoluble material was observed at early time points, 60 minutes, with larger aggregates not being able to penetrate into the separation gel. This insoluble material appeared to increase at the expense of soluble oligomers and monomers. At longer fibrillation times, 180 minutes, the presence or absence of curcumin did not result in any difference in the amount of large aggregates nor oligomers or monomers. Even though TEM is not a quantitative method, TEM analysis was performed to obtain a morphological assessment of how curcumin influenced Aβ_1–42_ aggregation. Micrographs obtained for Aβ_1–42_ incubated for 60 minutes in the absence of curcumin displayed a great amount of morphologically disordered aggregates, as well as few amyloid fibrils associated with the aggregates. In the presence of curcumin, even at the lowest concentration, it appeared that the amount of fibrils was enhanced. At 180 min, large networks of entangled fibrils were present in all samples. Taken together, the PAGE and TEM analyses showed that curcumin does not inhibit fibrillation of Aβ_1–42_, but on the contrary appeared to enhance fibrillation. A fluorescence based assay, using p-FTAA as a probe for formation of prefibrillar aggregates (including amyloid oligomers) and amyloid fibrils [29], [35], was also performed. Conducting this assay in the presence of curcumin is difficult, because curcumin re-absorption of light will decrease the fluorescence signal and also competition between binding sites can occur. Hence, at least to accommodate the first reservation the fluorescence intensity was normalized for each curcumin concentration. As shown previously, p-FTAA fluoresces strongly in the presence of prefibrillar aggregates, and in the presence of only vehichle (2% EtOH) Aβ_1–42_ rapidly forms p-FTAA positive aggregates with a plateau at 60–240 min, followed by a decreased signal (Figure 7D and Figure S7). Interestingly, the presence of curcumin appeared to suppress the formation of the initial prefibrillar phase, in a concentration dependent manner. These results support the notion that curcumin shifts the Aβ_1–42_ fibrillation pathway away from prefibrillar aggregates/oligomers and towards amyloid fibrils.

## Discussion

In the present work we aimed to formulate a pharmacological treatment study of *Drosophila* AD models. The compound curcumin was selected from its well documented ability to be non-toxic, to mitigate neuropathology and to prevent cytotoxic Aβ aggregate accumulation. A recent study by Alavez and colleagues in *Caenorhabditis elegans* shows the promise of curcumin (and Thioflavine T) as a drug candidate towards protein misfolding during aging and Aβ_3–42_ amyloidosis [36]. Our findings in *Drosophila* are rather different than that reported in the Alavez study. In *C. elegans*, no toxic effects were found on control worms, but rather a life enhancing effect. Furthermore, curcumin treated worms exhibited a decreased amount of Aβ accumulation. The mechanistic outcome from our study was hence quite different even though the message is the same: curcumin prolongs lifespan and reduces neurodegeneration in an invertebrate model system for Aβ amyloidosis.

As anticipated from previous reports on the potency of curcumin, the lifespan and activity behavior of AD fly models treated with curcumin displayed a positive pharmacological effect. Unexpectedly, control flies exhibited a substantially reduced survival as a function of curcumin concentration. Importantly, the pharmacological effect was directly dependent on genotype, rendering the strongest positive curcumin effect on Aβ expressing flies exhibiting the worst phenotype. The curcumin induced pharmacological effect was in the following order: Aβ_1–40_<single insert Aβ_1–42_<double insert Aβ_1–42_<Aβ_1–42 E22G_. Intriguingly the reverse order of curcumin toxicity was evident: control>Aβ_1–40_>single insert Aβ_1–42_>double insert Aβ_1–42_>Aβ_1–42 E22G_. This became most evident when comparing the median life span of the different genotypes as a function of curcumin concentration (Figure S8). Hence the marked toxicity of curcumin for *Drosophila* controls was decreased or totally depleted for the amyloid expressing trangenes, indicating that Aβ (and possibly also Tau) can function as a chemical detoxifier. If this is a normal function of Aβ amyloid is not known, but, in the context of other Aβ xenobiotic systems, a copper detoxification role within the context of transgenic *Caenorhabditis elegans* was recently reported [37]. We cannot rule out effects on other molecular pathways compared to direct binding to Aβ, however we have shown that Aβ_1–42_ does bind curcumin *in vitro*. It is therefore particularly interesting that Aβ appeared to suppress curcumin induced toxicity in the flies. It is hence possible that curcumin metabolites are responsible for the induced toxicity noted in the fly, and that this toxic effect was hindered by its sequestration within Aβ.

The increased lifespan upon curcumin treatment was especially striking for flies expressing the Aβ peptide with the so called Arctic mutation; Aβ_1–42 E22G_. The Aβ_1–42 E22G_ expressing flies increased their median survival with a striking 75% for the intermediate curcumin concentration. In the survival assay no positive effect on curcumin treatments was observed for Tau expressing flies, but rather a toxic effect at the highest curcumin concentration. Nevertheless the flies expressing Tau showed sustained locomotor activity and the largest increase in activity of all genotypes upon curcumin treatment at the intermediate concentration. This enhanced activity was sustained over time, in contrast to treatment of *Aβ* transgenes, suggesting a different mechanism, possibly related to decreased oxidative stress [38], [39] arising from antioxidant activity of curcumin.

Histological staining of fly brains followed by visual inspection showed that the treatment with curcumin at the intermediate concentration appeared to accelerate the formation of Aβ amyloid fibrils (p-FTAA positive), as opposed to those reactive with Aβ specific antibody. This was evident by analysis of the protein aggregation progression by microscopy at different ages (Table 1). At later time points (close to the day of death) the difference was less pronounced, but most importantly no decrease in total amount of amyloid deposition was observed as a function of curcumin treatment. In this fly model >95% of accumulated Aβ_1–42_ is insoluble, and a redistribution of soluble/insoluble Aβ_1–42_ can only be quantified if the soluble fraction was to increase during curcumin treatment. Our data showed that curcumin treatment did not affect the amount or the soluble/insoluble distribution of Aβ deposition as quantified by the MSD immunoassay. Furthermore, aged flies expressing the Aβ_1–42_ peptides in the eyes by a strong driver (*GMR-Gal4* crossings), analyzed by histological staining in the fluorescence microscope after staining with antibody, the LCO, and the nuclear marker DAPI, also displayed indifferent amyloid deposition patterns upon curcumin treatment (Figure S9). Taken together, in neither of our experiments did we observe any decrease in the amount of Aβ or Tau deposition following curcumin treatment.

**Figure 8:**
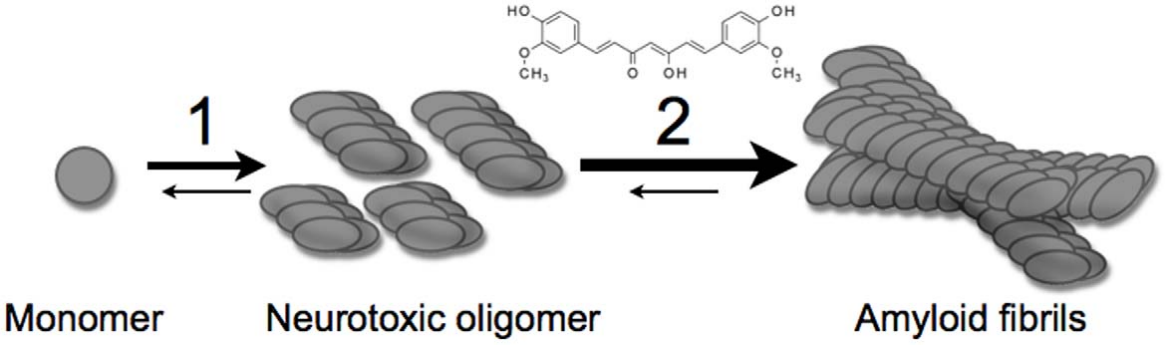
Schematic illustration of Aβ aggregation in *Drosophila*. The aggregation from soluble Aβ monomer to Aβ oligomers (step 1) and further maturation to Aβ amyloid fibrils (step 2) is dependent on age. The Arctic mutation (E22G) favors the formation of oligomers. In the presence of curcumin, the aggregation into Aβ amyloid fibrils (step 2) is favored.

One striking finding in this study was the hyper spectral analysis of p-FTAA fluorescence over time performed on double insert Aβ_1–42_ expressing flies: curcumin treatment accelerated the formation of well ordered amyloid fibrils in middle age. The notion that curcumin accelerated conformational conversion, rather than modulating the levels of Aβ, was hence supported by the quantification of similar amounts of Aβ in both treated and untreated brains. Our observations support recent findings in transgenic AD mouse models [20] and *in vitro* experiments [40], where it has been suggested that curcumin modulates the Aβ amyloid cascade by accelerating fibril formation and decreasing oligomer formation (Figure 8). Fibrillation assays of recombinant Aβ_1–42_ peptide in absence and presence of curcumin was performed in order to verify such activity of the curcumin used in our *Drosophila* assay. The *in vitro* fibrillation assays of recombinant Aβ_1–42_ peptide in our study hence confirmed the few previous reports that curcumin decrease the population of soluble oligomers and appeared to accelerate formation of large amyloid fibrils in contrast to the majority of reports stating the fibrillation inhibitory mechanism for curcumin.

The Arctic mutant peptide Aβ_1–42 E22G_ is well established to form soluble oligomeric protofibrils with high cytotoxicity [41], and is likely the reason for its strong toxicity in *Drosophila* [3], [5]. The finding that sub-stoichiometric amounts of curcumin stabilize nucleation of fibril formation *in vitro* correlates very well with the notion that reduced oligomerization and increased fibril formation in the presence of curcumin markedly protects the Aβ_1–42 E22G_ expressing flies. Our findings in the fly correlates well with those previously reported using mutagenesis where one additional mutation Aβ_1–42 I31E_ in the context of Aβ_1–42 E22G_, mitigated neurotoxicity in *Drosophila* by enhancement of amyloid fibril conversion at the expense of oligomers [42]. The same mechanism could explain the neuroprotective effect of curcumin seen for the wild type Aβ expressing flies.

### Concluding remarks and outlook

Expressing Aβ in the *Drosophila* CNS results in a significant neurotoxic effect, in contrast to that observed in mouse models of AD, which indicates the potency of these models for pharmacological treatment studies.We have demonstrated that curcumin exerts a general neuroprotective effect for *Aβ* and *tau Drosophila* transgenes. We have demonstrated that this curcumin activity in the context of Aβ is due to accelerated fibril conversion at the expense of putatively neurotoxic oligomers. It is plausible that the apparent toxicity of curcumin within *Drosophila*, which appears to be absent for mammalian cells, does suggest that the neuroprotective effect of curcumin can be even stronger than that reported here. The main drawback for curcumin as a drug for treatment of AD appears to be the poor bioavailability and stability in solution. With that in mind it is encouraging that curcumin analogues are synthesized as candidate drugs towards AD [43].

## Materials and Methods

### Fly stocks

*Drosophila* stocks were allowed to develop under a 12∶12 hours light∶dark cycle until eclosion at 26°C and posteclosion at 29°C, at 70% humidity. The flies were kept in 50 ml plastic vials containing standard *Drosophila* food (corn meal, molasses, yeast, and agar) and maintained post eclosion in 50 ml plastic vials containing 7 ml agar (20 g agar, 20 g sugar dissolved in 1 l of milliQ water) and yeast paste (dry bakery yeast dissolved in milliQ water) at 29°C under 12∶12 hours light-dark cycles, in a setup of twenty flies per vial. The vials were changed every second day. Curcumin (28260, Fluka) was dissolved in 95% ethanol to a concentration of 10 mg/ml and then diluted into the yeast paste into a final concentration of 1, 10 and 100 µg curcumin per g yeast paste, corresponding to (w/w) 0.0001%, 0.001%, and 0.01% curcumin added. Even distribution of curcumin within the yeast paste was easily checked by visual inspection due to the strong yellow color of the compound. All three curcumin concentrations were used in the lifespan and climbing assays, but only the intermediate concentration of 10 µg curcumin per g yeast paste was used in the activity assay, for flies corresponding to the immunohistochemistry combined with LCO staining, and for flies corresponding to quantification of Aβ levels by MSD. The *C155-Gal4* driver [44] was used for all experiments except for the eye degeneration assays were the *GMR-Gal4* driver [45] was used instead. The UAS transgenes used in the experiments were *Aβ_1–40_* (*UAS-Aβ_1–40_*), single insert *Aβ_1–42_* (*UAS-Aβ_1–42_*), double insert *Aβ_1–42_* (*UAS-Aβ_1–42_;UAS-Aβ_1–42_*), *Aβ_1–42 E22G_* (*UAS-Aβ_1–42 E22G_*) [3], and *tau* (*UAS-tau*) [1].

### Survival Assay

The survival assay is a standard assay for monitoring the effect of genotype or environmental conditions on *Drosophila* lifespan, and is especially useful for *C155-Gal4* lines expressing toxic proteins in the CNS. The number of surviving flies corresponding to the *C155-Gal4* crossings was counted every second day. The survival proportions were calculated using the Kaplan-Meier estimation [46] by using the log-rank method in the GraphPad Prism 5.0 d software (GrapPad Software Inc., San Diego, CA, USA). The survival times described in the study are given as median ± standard error of the median. The number of flies in the assay was between 100 and 60 flies of each genotype, as specified in Table S2. Significance was compared using a paired student's t-test between untreated (0% curcumin) and treated with curcumin (*: P<0.05, **: P<0.01; ***: P<0.001).

### *Drosophila* Locomotor Activity Assay

The activity of individual flies (n = 16) corresponding to the *C155-Gal4* crossings was recorded using *Drosophila* Activity Monitors (DAM2, TriKinetics, Waltham, MA, USA), and the number of beam breaks per hour during 24 hours was registered. The DAM2 unit was placed with a light source at the front of the monitor. The assay started with 10 hours of light followed by 12 hours in the dark, and 2 hours of light. The activity was measured at 5, 10, 15, and 20 days after eclosion for all genotypes and curcumin treatments, except for Aβ_1–42 E22G_ expressing flies and the Tau expressing flies, which was only measured for 5–10 and 5–15 days, respectively, due to their shortened lifespan. The total number of beam breaks were calculated for all genotypes and curcumin treatments and compared using two-tailed student's t-test of unpaired samples (*: P<0.05, **: P<0.01; ***: P<0.001) with GraphPad Prism 5.0 d software (GrapPad Software Inc., San Diego, CA, USA).

### Histological Analysis of Amyloid

Mildly fixed sections of *Drosophila* brains were assayed by histological staining for the presence of amyloid deposits using a combination of immunofluorescence and the amyloid specific luminescent conjugated oligothiophene (LCO), p-FTAA (*4′,3‴-Bis-carboxymethyl-[2,2′;5′,2″;5″,2‴;5‴,2″″]quinquethiophene-5,5″″-dicarboxylic acid*) [29]. The amyloidotropic p-FTAA is a conformational-dependent probe with a flexible structure that can serve as a surrogate marker to reveal heterogeneity among amyloid deposits in a manner analogous to that reported for luminescent conjugated polythiophenes (LCP) [47], [48], [49]. Newly eclosed, ten, and twenty day old flies corresponding to the *C155-Gal4* and newly eclosed and twenty day old flies corresponding to the *GMR-Gal4* crossings were mounted in Tissue-Tek® O.C.T. Compound (4583, Histolab, Sweden). The *C155-Gal4* crossings were used to study amyloid aggregation over lifetime, and the *GMR-Gal4* crossings were used to study late stages of amyloid aggregation independent of genotype lifespan. The fly heads were sectioned and stained during standard procedures [32]. The *C155-Gal4/UAS-Aβ_1–42 E22G_* and *C155-Gal4/UAS-tau* were mounted at day 0, day 5, and 10 and at day 0, day 10, and 15 respectively due to their shortened lifespan. The late time points approximately correspond to their T_1/2_, and the earlier time point approximately correspond to half of the time for their T_1/2_. For protein detection monoclonal mouse anti-β-Amyloid (3740-5-100, Mabtech, Sweden) diluted 1∶200 in 4% BSA/PBST (PBS with 0.1% Triton-X 100) and monoclonal mouse anti-human PHF-Tau AT8 (MN1020, Pierce Endogen) diluted 1∶100 in 4% BSA/PBST and visualized using donkey anti-mouse Alexa Fluor® 594 (A21203, Invitrogen Molecular Probes) diluted 1∶500 in 4% BSA/PBST. As amyloid specific probe the LCO; p-FTAA [29], diluted 1∶500 (from a 1 mg/ml stock) in PBS was used. The sections were stained by the nuclear marker DAPI by using Vectashield*®* with DAPI (H1200, Vector laboratories, Burlingame, CA, USA) as mounting medium. An Inverted fluorescence microscope (Carl Zeiss Inc.), with 405/30 nm, 470/40 and 546/12 nm longpass filter or a fluorescence microscope (Leica Microsystems Inc., Bannockburn, IL, USA), with 405/40 nm, 436/20 and 560/40 nm longpass filter, both attached with a spectral camera (Applied Spectral Imaging Inc.) were used to obtain the micrographs.

At least 20 fly heads of each genotype that had been fed normal food or food containing 0.001% w/w curcumin (10 µg per gram of yeast) were assayed. An estimation of the aggregate load in each genotype was done by visual inspection in the fluorescence microscope.

### Spectral analysis of amyloid produced in *Drosophila*

Consecutive sections to the histological analyses was used for protein spectral analyzes by the use of the LCO; p-FTAA [29], [32]. The p-FTAA was diluted 1∶500 (from a 1 mg/ml stock solution) in PBS and was used to stain sections as described before [32]. A fluorescence microscope (Leica Microsystems Inc., Bannockburn, IL, USA), with 405/40 nm and 560/40 nm longpass filter, attached with a spectral camera (Applied Spectral Imaging Inc.) was used to collect spectral images in an interval of 450 to 700 nm. LCO hyper spectral microscopy analysis of *Drosophila* brain aggregates was used to evaluate the conformation of deposited Aβ and Tau. The ratio of the emission peak at 508 nm and the emission peak at 612 nm for the LCO, p-FTAA, taken at 405 nm excitation and 560 nm excitation (Figure S3) was plotted as an amyloid fibrillation index. Spectral analysis of the two excitation spectra was performed by the SpectraView® software (Applied Spectral Imaging Inc.) and were further analyzed by the GraphPad Prism 5.0 d software (GrapPad Software Inc., San Diego, CA, USA). The spectral shift of the LCO was analyzed by the fraction of the blue shifted peak (at 508 nm from the 405 nm excitation) and the red shifted peak (at 612 nm from the 560 nm excitation). The spectral shifts were estimated as the medium fraction with the standard deviation and compared using two-tailed student's t-test of unpaired samples (*: P<0.05, **: P<0.01; ***: P<0.001).

To distinguish between different forms of aggregates formed in the double inserted Aβ_1–42_ expressing flies corresponding to the *C155-Gal4* crossing in absence or presence of curcumin at different ages, the fraction of the peaks at 508 nm and 543 nm verses the peaks at 508 nm and 612 nm were analyzed by the GraphPad Prism 5.0 d software (GrapPad Software Inc., San Diego, CA, USA). The spectral shifts were estimated as the medium fraction with the standard deviation and compared using two-tailed student's t-test of unpaired samples (*: P<0.05, **: P<0.01; ***: P<0.001).

At least five different brains of the different genotypes at different time points were analyzed. The number of spectra taken was dependent on amount of amyloids detected. At least 15 spectra were collected of every genotype at the different time points.

### Quantification of the Aβ_1–42_-peptide in aged *Drosophila*

Five fly heads of newly eclosed, ten and twenty day old double inserted Aβ_1–42_ expressing flies and newly eclosed, five and ten day old Aβ_1–42 E22G_ expressing flies, both corresponding to the *C155-Gal4* crossing, were homogenized in 50 µl of extraction buffer (50 mM Hepes pH 7.3, 5 mM EDTA, Protease inhibitor (Complete™, Roche Diagnostics)). The homogenate was incubated at room temperature for 10 min, sonicated for 4 minutes in a water bath and then centrifuged at 12 000 *g* for 5 minutes into a “soluble” and “insoluble” fraction. 20 µl of the supernatant (“soluble fraction”) was mixed with 180 µl hepes dilution buffer (25 mM Hepes pH 7.3, 1 mM EDTA, 0,1% MSD Blocker A (R93BA-4, Meso Scale Discovery, MD, USA)). The pellet (“insoluble fraction”) was homogenized in 50 µl of extraction buffer containing guanidinium HCl (5 M GnHCl, 50 mM Hepes pH 7.3, 5 mM EDTA, Protease inhibitor (Complete™, Roche Diagnostics)). 20 µl of the supernatant of the “insoluble fraction” was mixed with 180 µl hepes dilution buffer prior to analysis. All homogenate samples were assayed in triplicates at three independent assay occasions.

The quantification of Aβ-peptides in the “soluble” and “insoluble” fractions were performed using the MSD® 96-Well MULTI-ARRAY® Human (6E10) Abeta 42 Ultra-Sensitive Kit (K151FUE-2, Meso Scale Discovery, MD, USA). Triplicate 25 µl aliquots of sample were mixed with an equal amount of MSD Blocker A (2% MSD Blocker A, 0.2% Tween 20 and protease inhibitor) and added to a MSD MULTI-SPOT® 96-well 4-spot plate. Detection was achieved by addition of 25 µl 1 µg/ml SULFO-TAG (R91AN-1, Meso Scale Discovery, MD, USA) 4G8 monoclonal antibody (SIG-39220-200, Nordic Biosite, Taby, Sweden) for *C155-Gal4/UAS-Aβ_1–42_;UAS-Aβ_1–42_*, or SULFO-TAG Anti Aβ42 antibody (D0011750, Meso Scale Discovery, MD, USA) for *C155-Gal4/UAS- Aβ_1–42_*_ E22G_, and measurements were taken in a SECTOR Imager 2400 instrument (Meso Scale Discovery, MD, USA). To adjust for variation in the protein extraction step a quantitation of the total amount of protein from each sample of fly homogenate was performed by usage of the Bio-Rad DC Protein Assay Kit II (500-0112, BioRad, CA, USA). The concentrations of Aβ were compared between untreated flies and flies treated with curcumin (10 µg per gram of yeast). Significance was calculated using two-tailed Student's t-test (*: P<0.05, **: P<0.01; ***: P<0.001) in the GraphPad Prism 5.0 d software (GrapPad Software Inc., San Diego, CA, USA).

### *In vitro* Fibrillation Assay

Recombinant Aβ_1–42_ HFIP (A-1163-2, rPeptide, Borgart, GA, USA) was dissolved in 2 mM NaOH into a concentration of 1 mg/ml (222 µM). The peptide was stored at −20°C. 10 µM Aβ_1–42_ HFIP was allowed to fibrillate in a 96-well assay plate (3915, Corning Inc., NY, USA) in 10 mM phosphate buffer pH 7.5 in the absence or presence of curcumin in concentrations of 0.27, 2.7, and 27 µM dissolved in EtOH (corresponding to (w/w) 0.0001%, 0.001%, and 0.01% added curcumin). The fibrillation was performed at 37°C, at 500 rpm. Aliquots were withdrawn at time points of 0, 60, and 180 minutes and were assayed by Western blotting (see below) and transmission electron microcopy (see below). The p-FTAA fluorescence assay was performed as described in [29].

### Native PAGE Western blotting

15 µl aliquots from the fibrillation assay at time points; 0, 60, and 180 minutes, were mixed with 15 µl Native sample buffer (161-0738, BioRad, CA, USA) and run on a 12% acrylamide gel during native condition. Pre-stained protein standards (161-0374, BioRad, CA, USA) and synthetic Aβ_1–42_ peptide were used to indicate the apparent molecular weights of peptides and aggregates. After electrophoresis, proteins were blotted onto Immobilon–P transfer membrane (IPVH00010, Millipore, Billerica, MA, USA) set at 100 V for 1 hours at room temperature. After transfer, membranes were rinsed in distilled water followed by blocking using 4% BSA/TBST (TBS with 0.1% Tween) for 2 hours at room temperature. Blocked membranes were incubated with Monoclonal mouse anti-Amyloid β_1–16_ (6E10) antibody (9320-02, Signet Laboratories Inc., Dedham, MA, USA) diluted 1∶10 000 in 4% BSA/TBST for 1 hours at room temperature. After incubation with primary antibody, membranes were washed three times for 10 minutes in TBST, incubated with alkaline phosphatase-conjugate anti-mouse IgG (ab6729-1, AbCam, Cambridge, MA, USA), diluted 1∶1 000 in 4% BSA/TBST for 30 minutes at room temperature, and washed again three times for 10 minutes in TBST. The membrane was developed using Immun-Star™ Chemiluminescent (170-5012, BioRad, CA, USA) and visualized using a LAS-400mini attached with a CCD- camera (Fujifilm corporation, Greenwood, SC, USA). The *in vitro* fibril formation assay was repeated at three different occasions with fresh solutions.

### Transmission Electron Microscopy

5 µl aliquots from the fibrillation assay at time points; 0, 60, and 180 minutes, were applied on formvar and carbon coated copper grids for transmission electron microscopy (Carbon-B copper grids, Ted Pella Inc., Pedding, CA, USA). The sample was allowed to adhere to the grid for two minutes at room temperature. The grid was washed with dH_2_O and incubated for 20 seconds with 5 µl 1% uranyl acetate. Transmission electron micrographs were taken using a Jeol-1230 electron microscope operating at 100 kV.

## Supporting Information

Figure S1 **Climbing fraction trajectories of different transgenic** ***Drosophila*** **for different curcumin treatments.** (A) Control flies showed decreased climbing activity during aging upon curcumin treatment. (B) Curcumin treatment of Aβ_1–40_ expressing flies showed a positive effect for low curcumin concentration and a toxic effect for the high curcumin concentration. (C) Curcumin treatment of single insert Aβ_1–42_ expressing flies showed a positive effect for the intermediate curcumin concentration. (D) Curcumin treatment of the double insert Aβ_1–42_ expressing flies revealed weakly positive effect on the climbing behavior but was non-significant for the T_1/2 climbing_. (E) Aβ_1–42 E22G_ expressing flies showed an increased climbing ability for all curcumin concentrations. (F) Tau expressing flies showed no significant effect on the climbing behavior upon curcumin treatments. **Symbols:** No curcumin added represented with black lines, 1, 10, and 100 µg curcumin per g yeast paste represented in yellow, orange, and red lines respectively. (G) Median time of climbing fraction of all genotypes with no curcumin added represented in black bars, 1, 10, and 100 µg curcumin per g yeast paste represented in yellow, orange, and red bars respectively.
(TIF)

Figure S2 **Curcumin affects the brain amyloid deposition histology patterns as a function of** ***Drosophila*** **genotype.** Micrographs of fly brains taken with 100× objective showing fluorescence from cell nuclei by DAPI (blue), amyloid aggregates by p-FTAA (green) and Aβ by αAβ-antibody (red). (A–E) Aβ_1–40_ expressing flies with and without treatment with curcumin at day 0, day 10, and day 20, displayed small perinuclear amyloid deposits at day 20. Earlier time points show protein aggregates recognized by the antibody, and to a minimal extent with p-FTAA. (F–J) Single insert Aβ_1–42_ expressing flies exhibited strong amyloid staining by p-FTAA, predominantly surrounding the nuclei at day 20 as well as for curcumin treated flies at day 10. At day 0, and untreated flies at day 10 showed protein aggregates recognized by the antibody, and to some extent with p-FTAA. (K–O) Tau expressing flies at day 0, day 5, and day 10 displayed p-FTAA and anti-Tau-positive aggregates. The aggregates were mostly found in regions were no or few cell nuclei were visible. Filled arrowheads show p-FTAA positive (amyloid aggregates) and open arrows indicate αAβ or αTau-antibody positive (diffuse Aβ/Tau accumulation). Scale bars represent 20 µm.
(TIF)

Figure S3 **Hyperspectral imaging of** ***in vivo*** **formed Aβ aggregates over time.** Spectral analysis of aggregates performed as in Figure S6, of double insert Aβ_1–42_ flies at different ages. (A) Newly eclosed double insert Aβ_1–42_ flies have few and small aggregates detectable with p-FTAA with variable added emission spectra. The contribution from the 508 nm peak (405/40 excitation) was commonly smaller than from the 612 nm peak (560/40 excitation). (B) At day 10, the double insert Aβ_1–42_ flies shows extensive Aβ aggregation, detectable with p-FTAA. The spectra of the aggregates showed a high contribution of the 508 nm peak (405/40 excitation) and a low contribution of the 612 nm peak (560/40 excitation). The shoulder peak at 508 nm was clearly visible, but was consistently much lower than the peak at 543 nm. This is likely an indicator of immature fibrils. (C) At day 20, the double insert Aβ_1–42_ flies have large aggregates formed that are clearly recognized by the LCO with added a more distinct double peak at 508 and 543 nm, indicating well organized amyloid fibrils.
(TIF)

Figure S4 **2D spectral analysis of amyloid in double insert Aβ_1–42_ expressing** ***Drosophila*** **showing acceleration of fibrillation by curcumin ingestion.** The fraction of the emission peak at 508 nm and the emission peak at 543 nm and the fraction of the emission peak at 508 nm and the emission peak at 612 nm of the LCO, p-FTAA, taken at 405/40 nm excitation and 560/40 nm excitation was plotted as a 2D amyloid fibrillation index. (A) The variable emission spectra from newly eclosed flies, resulted in a wide variation in the 2D amyloid fibrillation index, interpreted as a wide variation in the morphology of the aggregates. (B) After ten days there was still a variation in aggregate spectra detected by the LCO in untreated flies but with a shift towards low 508/612 nm ratios. (C) After ten days of curcumin treatment, the spectra were less wide spread and shifted towards higher 508/612 nm ratios indicating more well ordered aggregate morphology. At day 20, both untreated (D) and curcumin treated (E) flies showed the grouped 2D amyloid fibrillation indexes indicating amyloid fibrils with the same morphological structure as those observed in curcumin treated day 10 flies.
(TIF)

Figure S5 **Binding control of curcumin to histological sections of double insert Aβ_1–42_ expressing** ***Drosophila*** **at day 20.** (A) Curcumin fed control flies (0.001%) only stained with DAPI as nuclei marker, shows no specific curcumin staining (D) Curcumin fed double insert Aβ_1–42_ flies (0.001%) only stained with DAPI as nuclei marker, shows no specific curcumin staining of amyloid. The integration time for the green channel (470/40) was set to maximum (3 s) to enhance the intensity for the channel. This produced an over-bleed from the DAPI staining into the green channel (open arrows). (B) Curcumin (0.01% in ethanol) staining as a histological marker of amyloid in unfed control flies combined with DAPI staining for nuclei marker and (E) double insert Aβ_1–42_ flies, shows spot like appearance from precipitated compound for both control flies and Aβ expressing flies (open arrow in B). (E) Some amyloid was detected in Aβ expressing flies (arrow), but the fluorescence emission was too low from the aggregates to confirm if the emitted light represent the bound curcumin to Aβ aggregates and were only detected as background in the 470/40 excitation filter. (C) Curcumin staining of unfed control flies followed by p-FTAAstaining combined with DAPI staining, shows no amyloid staining in controls but in (F) Aβ expressing flies. Since the curcumin histological staining increased the background staining of the samples, the large aggregates were still detected by the LCO probe (arrow head), but smaller aggregates surrounding the nuclei's were more diffuse (arrow). The emission spectra from the 470/40 excitation showed typical p-FTAA spectra in (F). No excitation of the 405/30 filter was possible due to the DAPI staining, and the characteristic double peak of the p-FTAA was hence lacking.
(TIF)

Figure S6 **Spectral contribution for Aβ and Tau aggregates found in** ***Drosophila*** **.** A fluorescence microscope, with 405/40 nm and 560/40 nm longpass filter, attached with a spectral camera was used to collect hyper spectral images in an interval of 450 to 700 nm of aggregates found in brain tissue stained with the LCO, p-FTAA. Spectral additions of the two excitations was performed by the SpectraView® software. The spectral shift of the LCO was analyzed by the fraction of the peak at 508 nm from the 405/40 nm excitation setup, and the peak at 612 nm from the 560/40 nm excitation. For 2D analysis of the Aβ deposits, the peak at 543 nm, from the 405/40 nm excitation was used.
(TIF)

Figure S7 **Fluorescence based assay using p-FTAA as a probe for recombinant Aβ aggregation.** Raw data graph corresponding to Figure 7D in main article, with error bars represented SEM. No curcumin added (vehicle control 2% EtOH) represented with black lines, 0.0001, 0.001, and 0.01% (w/v) curcumin is represented in yellow, orange, and red lines respectively.
(TIF)

Figure S8 **Genotype dependent curcumin toxicity.** Normalized median survival time as a function of curcumin concentration. The data on the y-axis was calculated from the median survival time for each genotype without treatment (T_1/2 (0%)_) versus treatment with the respective curcumin concentration (T_1/2 (x%)_). Control flies (black), Aβ_1–40_ expressing flies (cyan), single insert Aβ_1–42_ expressing flies (blue), double insert Aβ_1–42_ expressing flies (green), Aβ_1–42 E22G_ expressing flies (red), and Tau expressing flies (magenta).
(TIF)

Figure S9 **Histological sections of** ***Drosophila*** **eyes co-stained with p-FTAA and antibody.** Micrographs of 20 day old *GMR-Gal4*/*UAS- Drosophila* eyes taken with 100× objective showing fluorescence from cell nuclei by DAPI (blue), amyloid aggregates by p-FTAA (green) and Aβ or Tau by αAβ or αTau-antibody (red). (A) Untreated and (B) curcumin treated control flies shown in exhibited no antibody or LCO binding species within the eye. Background staining was seen in the retina. (C) Aβ_1–40_ expressing flies and the same transgene treated with curcumin (D) showed small amyloid staining of LCO and antibody species surrounding the nuclei. (E) Single insert Aβ_1–42_ expressing flies showed strong amyloid staining with LCO, predominantly surrounding the nuclei. The aggregates propagated along the ommatidia. (F) The same level and location of amyloid deposits were observed in the curcumin treated single insert Aβ_1–42_ expressing flies. (G) Double insert Aβ_1–42_ expressing flies showed extensive amyloid staining including several long extended fibrillar aggregates. DAPI staining from regions with extensive LCO-positive amyloid structures was markedly decreased. (H) The same level and location of amyloid deposits were observed in the curcumin treated double insert Aβ_1–42_ expressing flies. (I) Aβ_1–42 E22G_ expressing flies showed spot-like staining from both LCO and the Aβ antibody, but exhibited a weaker LCO staining than that displayed for wild type single and double insert Aβ_1–42_ expressing flies. Some irregular nuclei were visible from the DAPI staining. (J) The same staining pattern was reveled for the curcumin treated Aβ_1–42 E22G_ expressing flies. (K) Tau expressing flies, and (L) the same transgene treated with curcumin, displayed extensive LCO and antibody-binding aggregates. The aggregates were mostly found in regions were no or few nuclei were visible. Scale bars represent 50 µm. Arrows indicate small aggregates and filled arrowheads indicate long extended fibrillar structures. Unfilled arrow head indicates large aggregates in areas with massive tissue degradation.
(TIF)

Table S1 **Median survival time (T_1/2_) in days for** ***C155-Gal4*** **crossings of genotypes with altered curcumin concentration.**
(DOCX)

Table S2 **Median time of successful climbing (T_1/2 climbing_) in days for** ***C155-Gal4*** **crossings of genotypes with altered curcumin concentration.**
(DOCX)

Table S3 **Mean concentration (pg) Aβ/(mg) total protein ± SEM for soluble and insoluble samples per fly.**
(DOCX)

Table S4 **Mean concentration (pg) Aβ/(mg) total protein ± SEM for soluble and insoluble samples per fly.**
(DOCX)
