# Supplementary figures and images for: Curcumin Promotes A-beta Fibrillation and Reduces Neurotoxicity in Transgenic Drosophila
Source: PLoS One. 2012 Feb 13;7(2):e31424. doi: 10.1371/journal.pone.0031424 (PMC3278449; doi:10.1371/journal.pone.0031424)

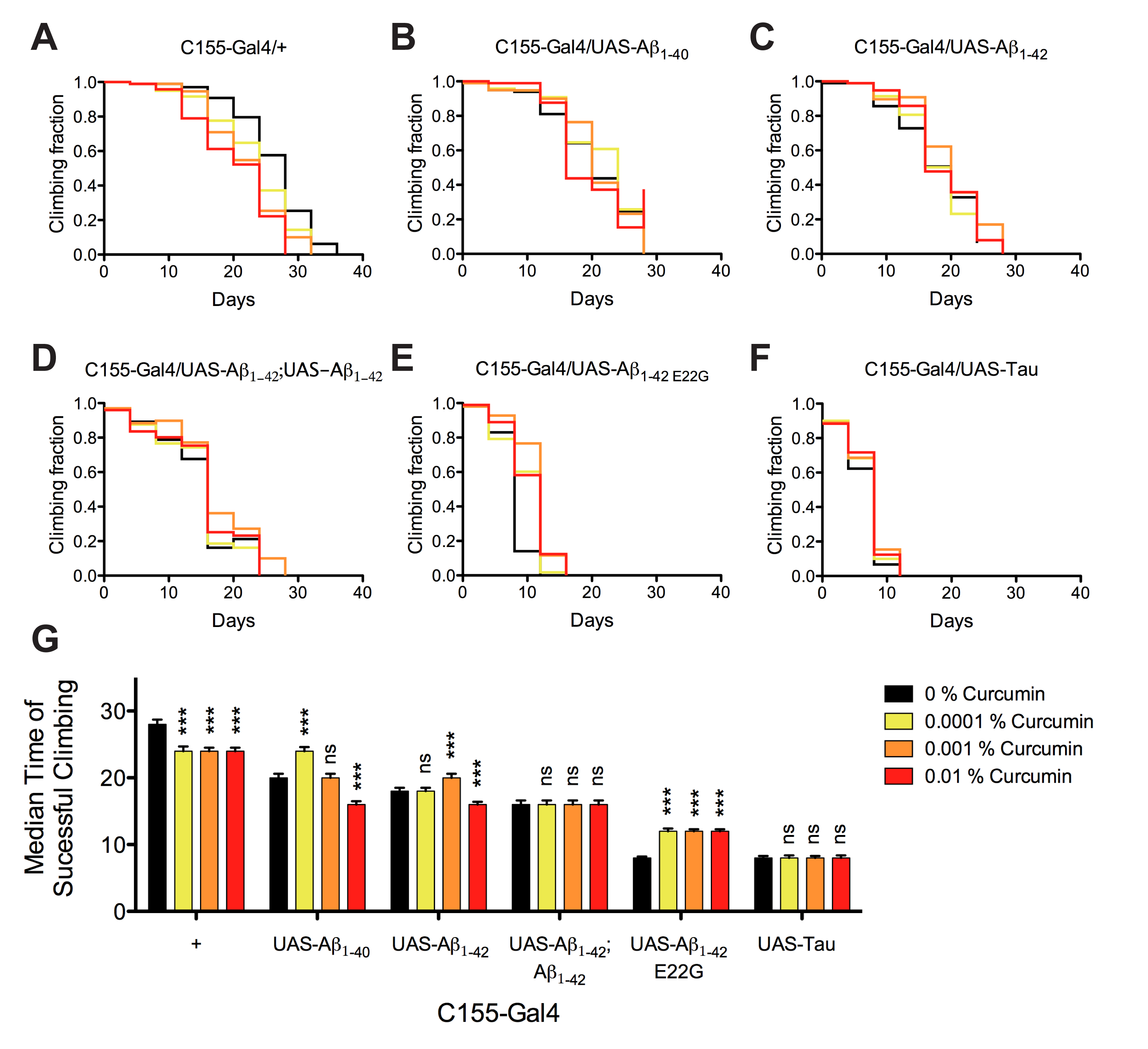

Supplement: Figure S1 — Climbing fraction trajectories of different transgenic Drosophila for different curcumin treatments. (A) Control flies showed decreased climbing activity during aging upon curcumin treatment. (B) Curcumin treatment of Aβ1–40 expressing flies showed a positive effect for low curcumin concentration and a toxic effect for the high curcumin concentration. (C) Curcumin treatment of single insert Aβ1–42 expressing flies showed a positive effect for the intermediate curcumin concentration. (D) Curcumin treatment of the double insert Aβ1–42 expressing flies revealed weakly positive effect on the climbing behavior but was non-significant for the T1/2 climbing. (E) Aβ1–42 E22G expressing flies showed an increased climbing ability for all curcumin concentrations. (F) Tau expressing flies showed no significant effect on the climbing behavior upon curcumin treatments. Symbols: No curcumin added represented with black lines, 1, 10, and 100 µg curcumin per g yeast paste represented in yellow, orange, and red lines respectively. (G) Median time of climbing fraction of all genotypes with no curcumin added represented in black bars, 1, 10, and 100 µg curcumin per g yeast paste represented in yellow, orange, and red bars respectively. (TIF) [file pone.0031424.s001.tif]

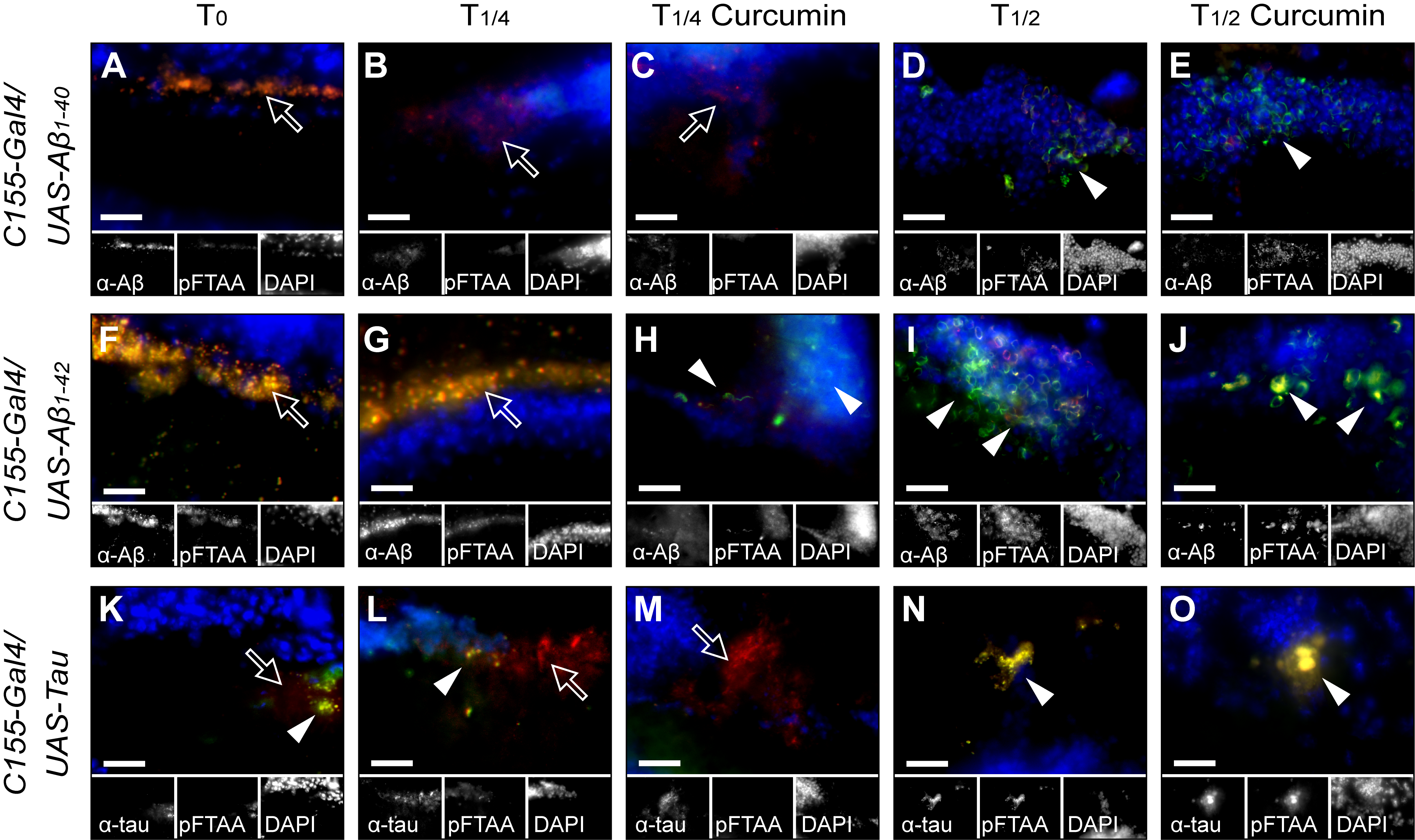

Supplement: Figure S2 — Curcumin affects the brain amyloid deposition histology patterns as a function of Drosophila genotype. Micrographs of fly brains taken with 100× objective showing fluorescence from cell nuclei by DAPI (blue), amyloid aggregates by p-FTAA (green) and Aβ by αAβ-antibody (red). (A–E) Aβ1–40 expressing flies with and without treatment with curcumin at day 0, day 10, and day 20, displayed small perinuclear amyloid deposits at day 20. Earlier time points show protein aggregates recognized by the antibody, and to a minimal extent with p-FTAA. (F–J) Single insert Aβ1–42 expressing flies exhibited strong amyloid staining by p-FTAA, predominantly surrounding the nuclei at day 20 as well as for curcumin treated flies at day 10. At day 0, and untreated flies at day 10 showed protein aggregates recognized by the antibody, and to some extent with p-FTAA. (K–O) Tau expressing flies at day 0, day 5, and day 10 displayed p-FTAA and anti-Tau-positive aggregates. The aggregates were mostly found in regions were no or few cell nuclei were visible. Filled arrowheads show p-FTAA positive (amyloid aggregates) and open arrows indicate αAβ or αTau-antibody positive (diffuse Aβ/Tau accumulation). Scale bars represent 20 µm. (TIF) [file pone.0031424.s002.tif]

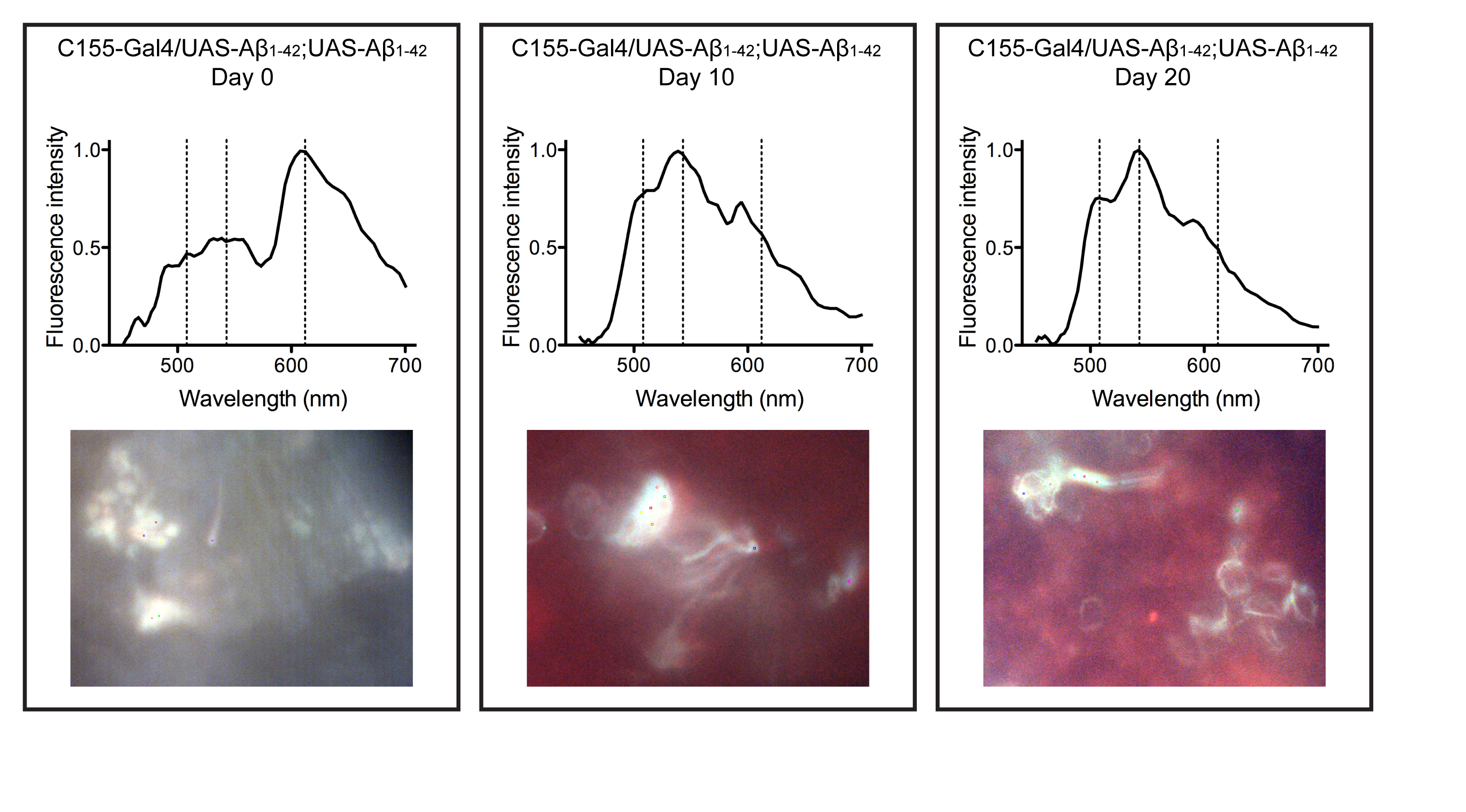

Supplement: Figure S3 — Hyperspectral imaging of in vivo formed Aβ aggregates over time. Spectral analysis of aggregates performed as in Figure S6, of double insert Aβ1–42 flies at different ages. (A) Newly eclosed double insert Aβ1–42 flies have few and small aggregates detectable with p-FTAA with variable added emission spectra. The contribution from the 508 nm peak (405/40 excitation) was commonly smaller than from the 612 nm peak (560/40 excitation). (B) At day 10, the double insert Aβ1–42 flies shows extensive Aβ aggregation, detectable with p-FTAA. The spectra of the aggregates showed a high contribution of the 508 nm peak (405/40 excitation) and a low contribution of the 612 nm peak (560/40 excitation). The shoulder peak at 508 nm was clearly visible, but was consistently much lower than the peak at 543 nm. This is likely an indicator of immature fibrils. (C) At day 20, the double insert Aβ1–42 flies have large aggregates formed that are clearly recognized by the LCO with added a more distinct double peak at 508 and 543 nm, indicating well organized amyloid fibrils. (TIF) [file pone.0031424.s003.tif]

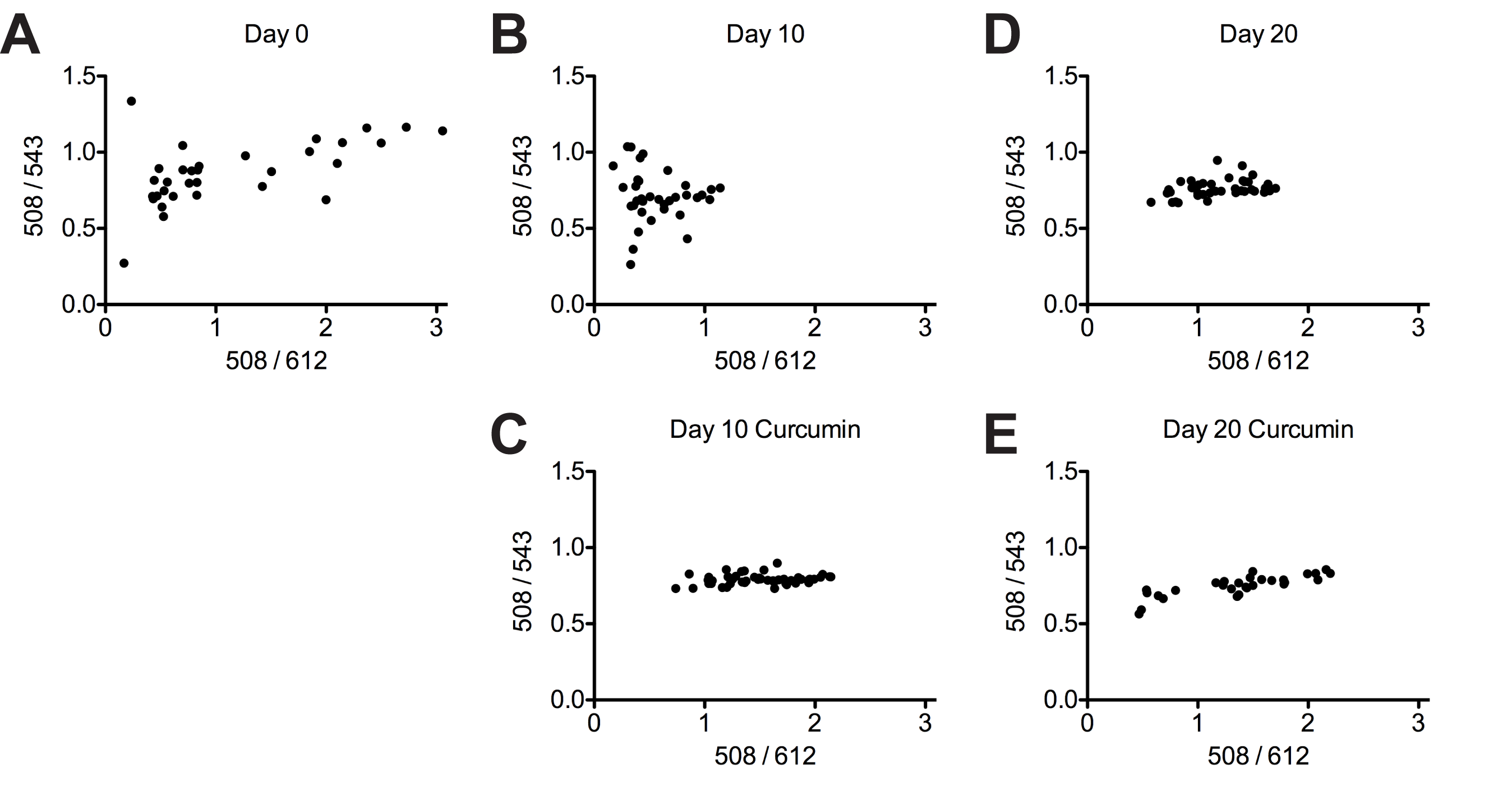

Supplement: Figure S4 — 2D spectral analysis of amyloid in double insert Aβ1–42 expressing Drosophila showing acceleration of fibrillation by curcumin ingestion. The fraction of the emission peak at 508 nm and the emission peak at 543 nm and the fraction of the emission peak at 508 nm and the emission peak at 612 nm of the LCO, p-FTAA, taken at 405/40 nm excitation and 560/40 nm excitation was plotted as a 2D amyloid fibrillation index. (A) The variable emission spectra from newly eclosed flies, resulted in a wide variation in the 2D amyloid fibrillation index, interpreted as a wide variation in the morphology of the aggregates. (B) After ten days there was still a variation in aggregate spectra detected by the LCO in untreated flies but with a shift towards low 508/612 nm ratios. (C) After ten days of curcumin treatment, the spectra were less wide spread and shifted towards higher 508/612 nm ratios indicating more well ordered aggregate morphology. At day 20, both untreated (D) and curcumin treated (E) flies showed the grouped 2D amyloid fibrillation indexes indicating amyloid fibrils with the same morphological structure as those observed in curcumin treated day 10 flies. (TIF) [file pone.0031424.s004.tif]

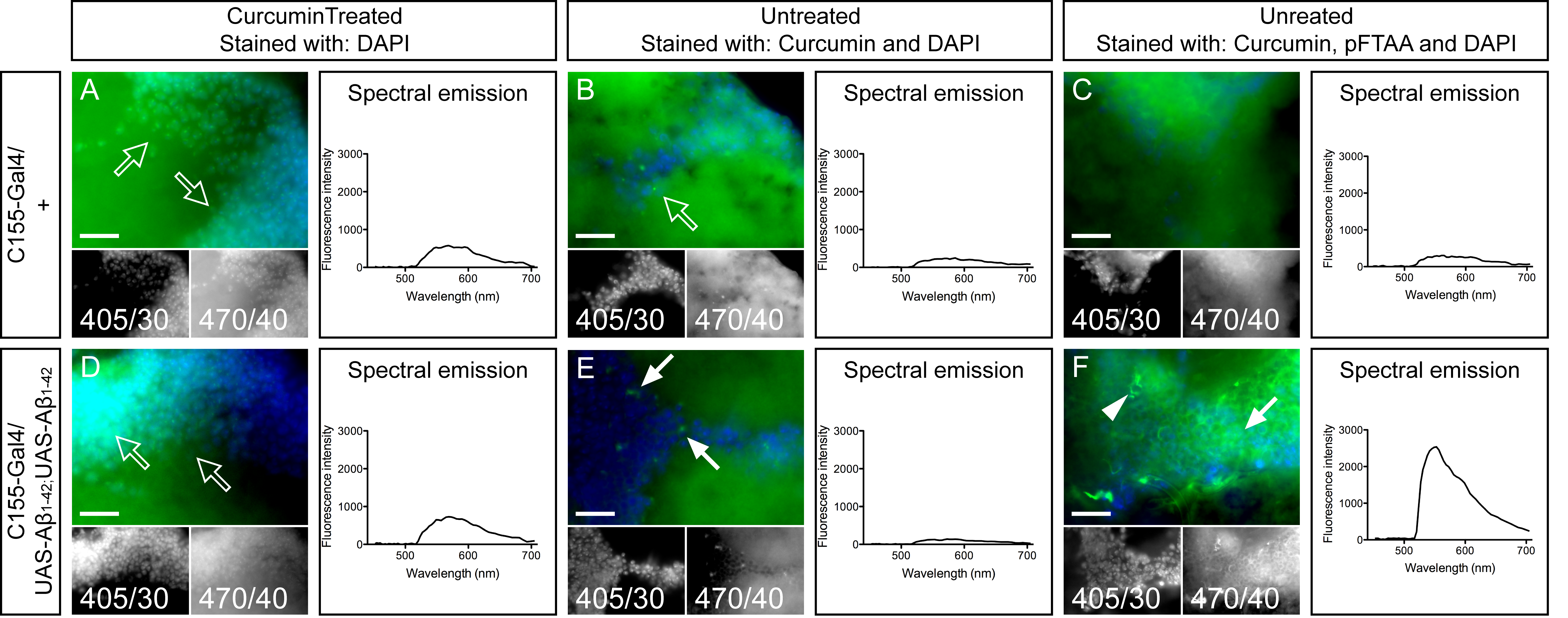

Supplement: Figure S5 — Binding control of curcumin to histological sections of double insert Aβ1–42 expressing Drosophila at day 20. (A) Curcumin fed control flies (0.001%) only stained with DAPI as nuclei marker, shows no specific curcumin staining (D) Curcumin fed double insert Aβ1–42 flies (0.001%) only stained with DAPI as nuclei marker, shows no specific curcumin staining of amyloid. The integration time for the green channel (470/40) was set to maximum (3 s) to enhance the intensity for the channel. This produced an over-bleed from the DAPI staining into the green channel (open arrows). (B) Curcumin (0.01% in ethanol) staining as a histological marker of amyloid in unfed control flies combined with DAPI staining for nuclei marker and (E) double insert Aβ1–42 flies, shows spot like appearance from precipitated compound for both control flies and Aβ expressing flies (open arrow in B). (E) Some amyloid was detected in Aβ expressing flies (arrow), but the fluorescence emission was too low from the aggregates to confirm if the emitted light represent the bound curcumin to Aβ aggregates and were only detected as background in the 470/40 excitation filter. (C) Curcumin staining of unfed control flies followed by p-FTAAstaining combined with DAPI staining, shows no amyloid staining in controls but in (F) Aβ expressing flies. Since the curcumin histological staining increased the background staining of the samples, the large aggregates were still detected by the LCO probe (arrow head), but smaller aggregates surrounding the nuclei's were more diffuse (arrow). The emission spectra from the 470/40 excitation showed typical p-FTAA spectra in (F). No excitation of the 405/30 filter was possible due to the DAPI staining, and the characteristic double peak of the p-FTAA was hence lacking. (TIF) [file pone.0031424.s005.tif]

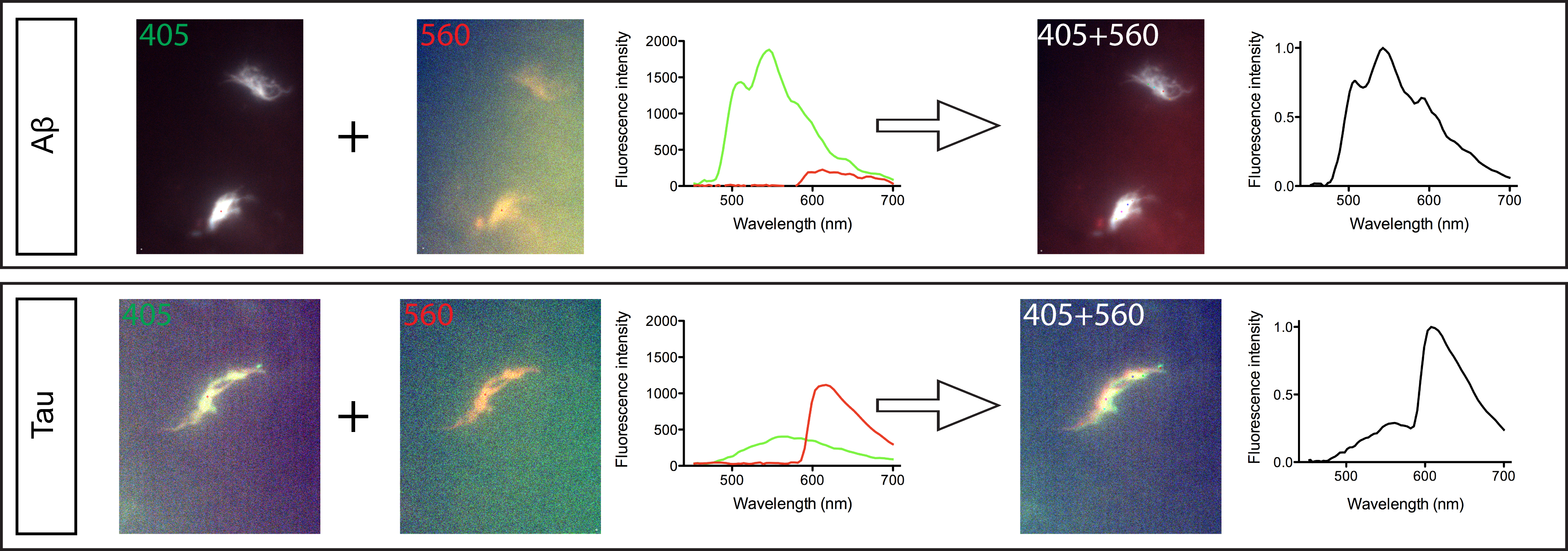

Supplement: Figure S6 — Spectral contribution for Aβ and Tau aggregates found in Drosophila . A fluorescence microscope, with 405/40 nm and 560/40 nm longpass filter, attached with a spectral camera was used to collect hyper spectral images in an interval of 450 to 700 nm of aggregates found in brain tissue stained with the LCO, p-FTAA. Spectral additions of the two excitations was performed by the SpectraView® software. The spectral shift of the LCO was analyzed by the fraction of the peak at 508 nm from the 405/40 nm excitation setup, and the peak at 612 nm from the 560/40 nm excitation. For 2D analysis of the Aβ deposits, the peak at 543 nm, from the 405/40 nm excitation was used. (TIF) [file pone.0031424.s006.tif]

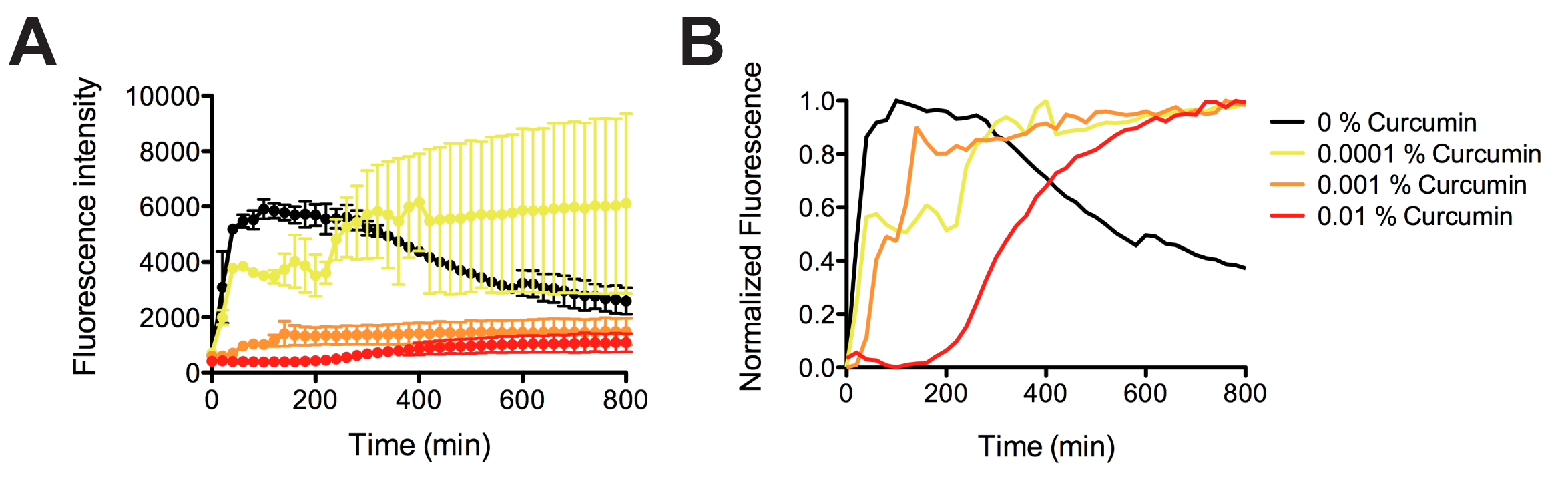

Supplement: Figure S7 — Fluorescence based assay using p-FTAA as a probe for recombinant Aβ aggregation. Raw data graph corresponding to Figure 7D in main article, with error bars represented SEM. No curcumin added (vehicle control 2% EtOH) represented with black lines, 0.0001, 0.001, and 0.01% (w/v) curcumin is represented in yellow, orange, and red lines respectively. (TIF) [file pone.0031424.s007.tif]

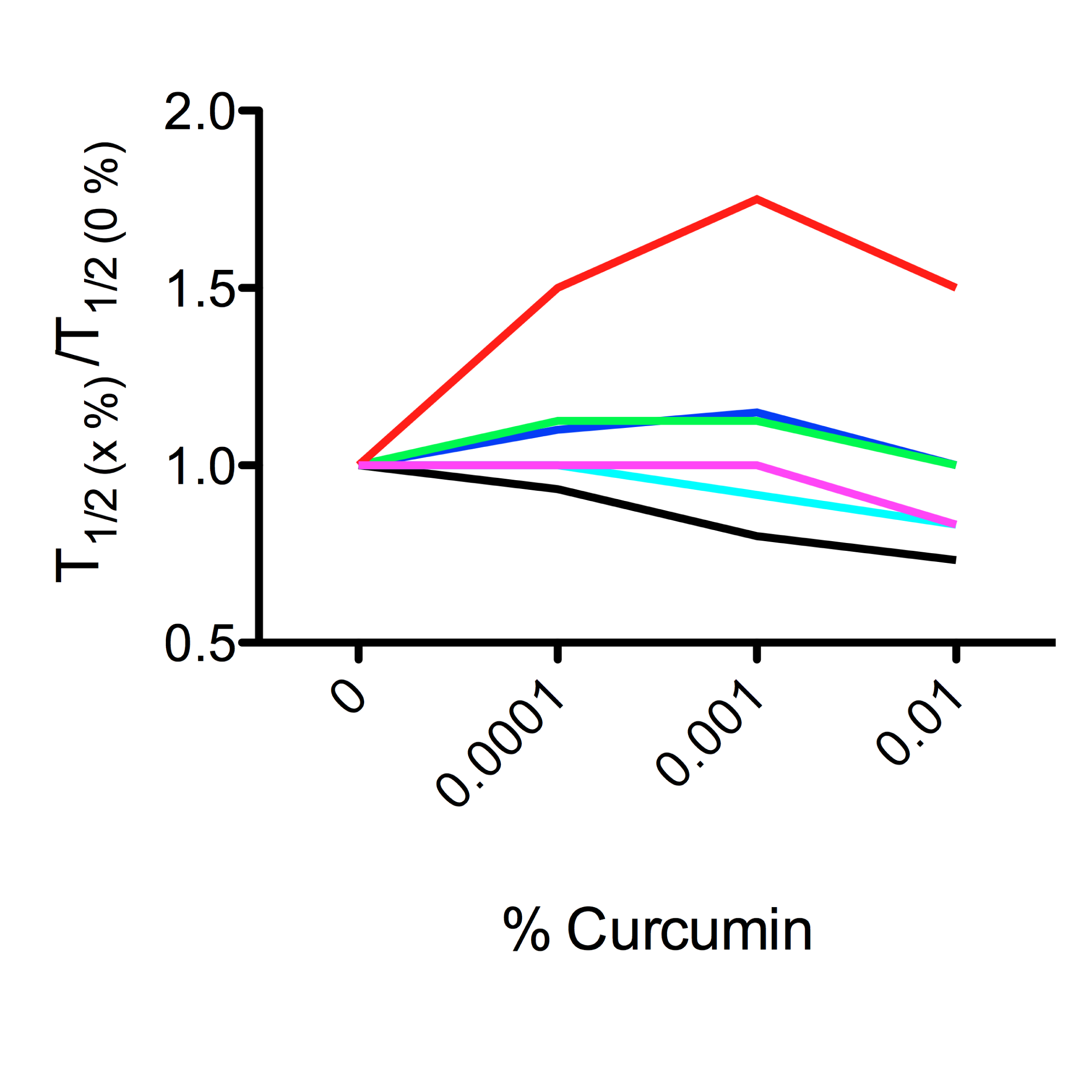

Supplement: Figure S8 — Genotype dependent curcumin toxicity. Normalized median survival time as a function of curcumin concentration. The data on the y-axis was calculated from the median survival time for each genotype without treatment (T1/2 (0%)) versus treatment with the respective curcumin concentration (T1/2 (x%)). Control flies (black), Aβ1–40 expressing flies (cyan), single insert Aβ1–42 expressing flies (blue), double insert Aβ1–42 expressing flies (green), Aβ1–42 E22G expressing flies (red), and Tau expressing flies (magenta). (TIF) [file pone.0031424.s008.tif]

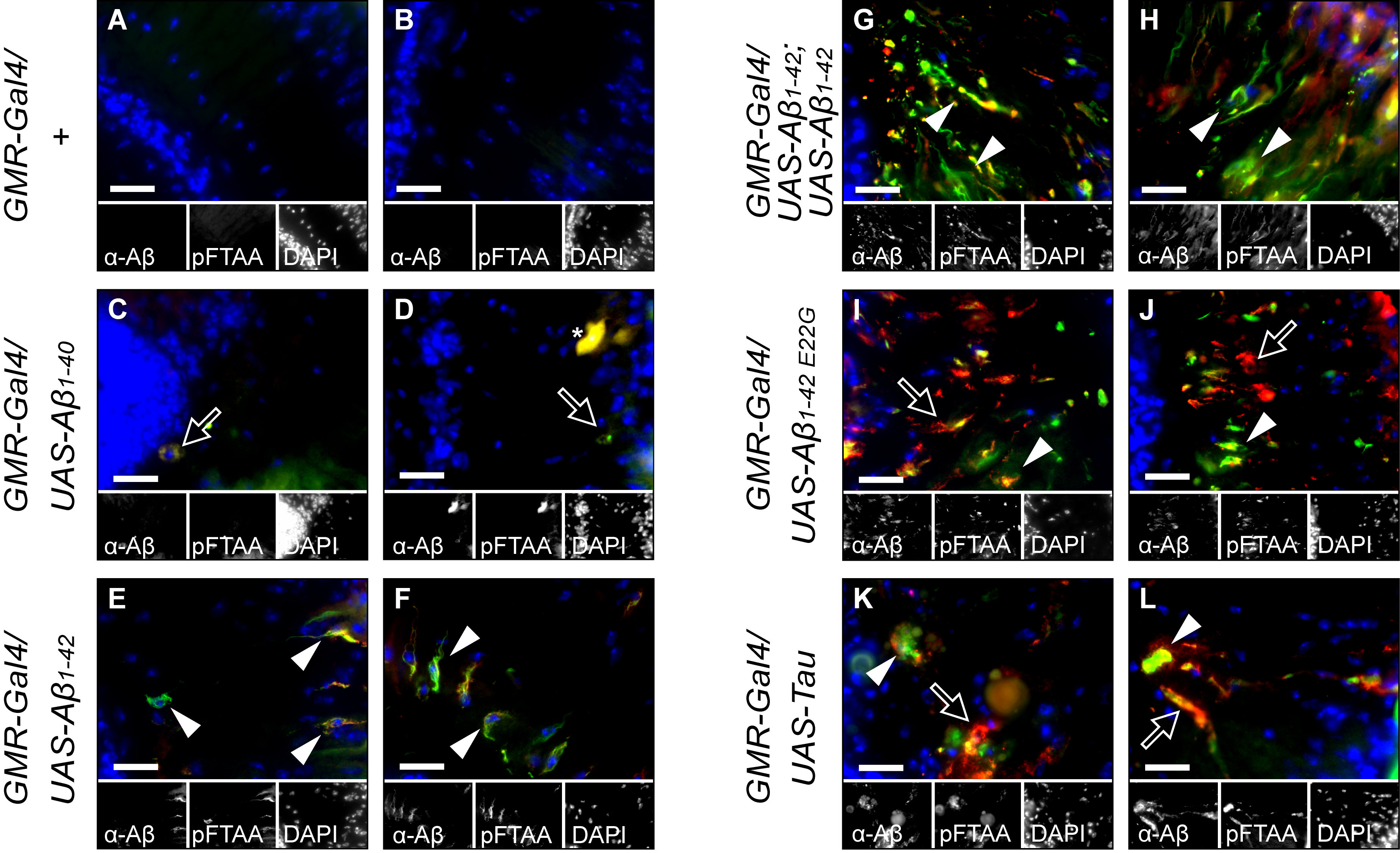

Supplement: Figure S9 — Histological sections of Drosophila eyes co-stained with p-FTAA and antibody. Micrographs of 20 day old GMR-Gal4/UAS- Drosophila eyes taken with 100× objective showing fluorescence from cell nuclei by DAPI (blue), amyloid aggregates by p-FTAA (green) and Aβ or Tau by αAβ or αTau-antibody (red). (A) Untreated and (B) curcumin treated control flies shown in exhibited no antibody or LCO binding species within the eye. Background staining was seen in the retina. (C) Aβ1–40 expressing flies and the same transgene treated with curcumin (D) showed small amyloid staining of LCO and antibody species surrounding the nuclei. (E) Single insert Aβ1–42 expressing flies showed strong amyloid staining with LCO, predominantly surrounding the nuclei. The aggregates propagated along the ommatidia. (F) The same level and location of amyloid deposits were observed in the curcumin treated single insert Aβ1–42 expressing flies. (G) Double insert Aβ1–42 expressing flies showed extensive amyloid staining including several long extended fibrillar aggregates. DAPI staining from regions with extensive LCO-positive amyloid structures was markedly decreased. (H) The same level and location of amyloid deposits were observed in the curcumin treated double insert Aβ1–42 expressing flies. (I) Aβ1–42 E22G expressing flies showed spot-like staining from both LCO and the Aβ antibody, but exhibited a weaker LCO staining than that displayed for wild type single and double insert Aβ1–42 expressing flies. Some irregular nuclei were visible from the DAPI staining. (J) The same staining pattern was reveled for the curcumin treated Aβ1–42 E22G expressing flies. (K) Tau expressing flies, and (L) the same transgene treated with curcumin, displayed extensive LCO and antibody-binding aggregates. The aggregates were mostly found in regions were no or few nuclei were visible. Scale bars represent 50 µm. Arrows indicate small aggregates and filled arrowheads indicate long extended fibrillar structures. Unfilled arro [file pone.0031424.s009.tif]
