# Supplementary material for: Curcumin Promotes A-beta Fibrillation and Reduces Neurotoxicity in Transgenic Drosophila
Source: PLoS One. 2012 Feb 13;7(2):e31424. doi: 10.1371/journal.pone.0031424 (PMC3278449; doi:10.1371/journal.pone.0031424)
Supplement: Table S1 — Median survival time (T1/2) in days for C155-Gal4 crossings of genotypes with altered curcumin concentration. (DOCX) [file pone.0031424.s010.docx]

**Table S1.** Median survival time (T_1/2_) in days for *C155-Gal4* crossings of genotypes with altered curcumin concentration.

| **Curcumin concentration (w/w)** | **control** | **Aβ_1-40_** | **Aβ_1-42_** | **Aβ_1-42_; Aβ_1-42_** | **Aβ_1-42 E22G_** | **tau** |
| --- | --- | --- | --- | --- | --- | --- |
| **0 %** | 30 ± 0.4 (n=200) | 24 ± 0.5 (n=200) | 20 ± 0.4 (n=200) | 16 ± 0.6 (n=100) | 8 ± 0.2 (n=180) | 18 ± 0.5 (n=80) |
| **0.0001 %** | 28 ± 0.6 (n=100) | 24 ± 0.7 (n=100) | 22 ± 0.5 (n=100) | 19 ± 0.5 (n=100) | 13 ± 0.5 (n=100) | 18 ± 0.6 (n=60) |
| **0.001 %** | 24 ± 0.5 (n=100) | 22 ± 0.5 (n=100) | 23 ± 0.6 (n=100) | 18 ± 0.6 (n=100) | 14 ± 0.5 (n=100) | 18 ± 0.5 (n=100) |
| **0.01 %** | 23 ± 0.6 (n=100) | 19 ± 0.6 (n=100) | 20 ± 0.5 (n=100) | 17 ± 0.5 (n=100) | 12 ± 0.3 (n=100) | 15 ± 0.7 (n=60) |
