# Supplementary material for: Curcumin Promotes A-beta Fibrillation and Reduces Neurotoxicity in Transgenic Drosophila
Source: PLoS One. 2012 Feb 13;7(2):e31424. doi: 10.1371/journal.pone.0031424 (PMC3278449; doi:10.1371/journal.pone.0031424)
Supplement: Table S2 — Median time of successful climbing (T1/2 climbing) in days for C155-Gal4 crossings of genotypes with altered curcumin concentration. (DOCX) [file pone.0031424.s011.docx]

**Table S2.** Median time of successful climbing (T_1/2 climbing_) in days for *C155-Gal4* crossings of genotypes with altered curcumin concentration.

| **Curcumin concentration (w/w)** | **control** | **Aβ_1-40_** | **Aβ_1-42_** | **Aβ_1-42_; Aβ_1-42_** | **Aβ_1-42 E22G_** | **tau** |
| --- | --- | --- | --- | --- | --- | --- |
| **0 %** | 28 ± 1.2 (n=100) | 22 ± 1.5 (n=100) | 18 ± 1.2 (n=100) | 16 ± 1.2 (n=100) | 8 ± 0.5 (n=100) | 8 ± 0.6 (n=80) |
| **0.0001 %** | 24 ± 1.4 (n=100) | 24 ± 1.5 (n=100) | 16 ± 1.2 (n=100) | 16 ± 1.2 (n=100) | 12 ± 0.7 (n=100) | 8 ± 0.8 (n=60) |
| **0.001 %** | 24 ± 1.1 (n=100) | 20 ± 1.2 (n=100) | 20 ± 1.2 (n=100) | 16 ± 1.4 (n=100) | 12 ± 0.5 (n=100) | 8 ± 0.9 (n=100) |
| **0.01 %** | 22 ± 1.3 (n=100) | 16 ± 1.2 (n=100) | 16 ± 1.0 (n=100) | 16 ± 1.3 (n=100) | 12 ± 0.5 (n=100) | 8 ± 0.8 (n=60) |
