# Supplementary material for: Curcumin Promotes A-beta Fibrillation and Reduces Neurotoxicity in Transgenic Drosophila
Source: PLoS One. 2012 Feb 13;7(2):e31424. doi: 10.1371/journal.pone.0031424 (PMC3278449; doi:10.1371/journal.pone.0031424)
Supplement: Table S3 — Mean concentration (pg) Aβ/(mg) total protein ± SEM for soluble and insoluble samples per fly. (DOCX) [file pone.0031424.s012.docx]

**Table S3**. Mean concentration (pg) Aβ / (mg) total protein ± SEM for soluble and insoluble samples per fly.

|  | **Day 0** | **Day 10** | | **Day 20** | |
| --- | --- | --- | --- | --- | --- |
| **Curcumin +/-** | **-** | **-** | **+** | **-** | **+** |
| **Soluble fraction^*^** | | | | | |
| *C155-Gal4/+* | 0.01 ± 0.011 | 0.01 ± 0.01 | 0.09 ± 0.09 | 0.15 ± 0.08 | 0.26 ± 0.14 |
| *C155-Gal4/UAS-Aβ_1-42_;UAS-Aβ_1-42_* | 3.03 ± 0.286 | 2.59 ± 0.26 | 3.29 ± 1.18 | 2.21 ± 0.55 | 1.58 ± 0.33 |
| **Insoluble fraction^**^** | | | | | |
| *C155-Gal4/+* | 1.65 ± 0.86 | 1.59 ± 0.92 | 1.57 ± 0.82 | 0.84 ± 0.42 | 1.21 ± 0.19 |
| *C155-Gal4/UAS-Aβ_1-42_;UAS-Aβ_1-42_* | 57.18 ± 12.04 | 124.76 ± 15.58 | 168.57 ± 28.81 | 149.23 ± 18.77 | 127.81 ± 31.13 |

^*^N=3 and ^**^N=5
