# Supplementary material for: Curcumin Promotes A-beta Fibrillation and Reduces Neurotoxicity in Transgenic Drosophila
Source: PLoS One. 2012 Feb 13;7(2):e31424. doi: 10.1371/journal.pone.0031424 (PMC3278449; doi:10.1371/journal.pone.0031424)
Supplement: Table S4 — Mean concentration (pg) Aβ/(mg) total protein ± SEM for soluble and insoluble samples per fly. (DOCX) [file pone.0031424.s013.docx]

**Table S4.** Mean concentration (pg) Aβ / (mg) total protein ± SEM for soluble and insoluble samples per fly.

|  | **Day 0** | **Day 5** | | **Day 10** | |
| --- | --- | --- | --- | --- | --- |
| **Curcumin +/-** | **-** | **-** | **+** | **-** | **+** |
| **Soluble fraction^*^** | | | | | |
| *C155-Gal4/+* | 0.28 ± 0.28 | 0.24 ± 0.24 | 0.22 ± 0.22 | 0.15 ± 0.15 | 0.46 ± 0.46 |
| *C155-Gal4/UAS-Aβ_1-42 E22G_* | 0.34 ± 0.34 | 1.87 ± 0.84 | 2.23 ± 0.37 | 2.01 ± 0.75 | 1.34 ± 0.38 |
| **Insoluble fraction^*^** | | | | | |
| *C155-Gal4/+* | 3.08 ± 2.34 | 2.56 ± 2.26 | 2.91 ± 2.60 | 2.59 ± 2.19 | 1.95 ± 1.71 |
| *C155-Gal4/UAS-Aβ_1-42 E22G_* | 161.11 ± 55.07 | 333.34 ± 68.47 | 246.05 ± 21.16 | 273.10 ± 79.87 | 312.40 ± 28.44 |

^*^N=3
